# A Comprehensive Review on Synergy of Multi-Modal Data and AI Technologies in Medical Diagnosis

**DOI:** 10.3390/bioengineering11030219

**Published:** 2024-02-25

**Authors:** Xi Xu, Jianqiang Li, Zhichao Zhu, Linna Zhao, Huina Wang, Changwei Song, Yining Chen, Qing Zhao, Jijiang Yang, Yan Pei

**Affiliations:** 1 Faculty of Information Technology, Beijing University of Technology, Beijing 100124, China; xuxi@bjut.edu.cn (X.X.); lijianqiang@bjut.edu.cn (J.L.); zhuzc@emails.bjut.edu.cn (Z.Z.); zhao_linna@emails.bjut.edu.cn (L.Z.); huinawang@emails.bjut.edu.cn (H.W.); songchangwei1996@emails.bjut.edu.cn (C.S.); chenyn0317@emails.bjut.edu.cn (Y.C.); 2 Tsinghua National Laboratory for Information Science and Technology, Tsinghua University, Beijing 100084, China; yangjijiang@tsinghua.edu.cn; 3 School of Computer Science and Engineering, The University of Aizu, Aizuwakamatsu 965-8580, Japan; peiyan@u-aizu.ac.jp

**Keywords:** multi-modal data, artificial intelligence, disease diagnosis, machine learning, deep learning, large model

## Abstract

Disease diagnosis represents a critical and arduous endeavor within the medical field. Artificial intelligence (AI) techniques, spanning from machine learning and deep learning to large model paradigms, stand poised to significantly augment physicians in rendering more evidence-based decisions, thus presenting a pioneering solution for clinical practice. Traditionally, the amalgamation of diverse medical data modalities (e.g., image, text, speech, genetic data, physiological signals) is imperative to facilitate a comprehensive disease analysis, a topic of burgeoning interest among both researchers and clinicians in recent times. Hence, there exists a pressing need to synthesize the latest strides in multi-modal data and AI technologies in the realm of medical diagnosis. In this paper, we narrow our focus to five specific disorders (Alzheimer’s disease, breast cancer, depression, heart disease, epilepsy), elucidating advanced endeavors in their diagnosis and treatment through the lens of artificial intelligence. Our survey not only delineates detailed diagnostic methodologies across varying modalities but also underscores commonly utilized public datasets, the intricacies of feature engineering, prevalent classification models, and envisaged challenges for future endeavors. In essence, our research endeavors to contribute to the advancement of diagnostic methodologies, furnishing invaluable insights for clinical decision making.

## 1. Introduction

The task of disease diagnosis holds significant importance within the medical domain. Timely diagnosis not only facilitates the prompt implementation of therapeutic interventions but also mitigates the risks associated with disease progression and complications, particularly concerning global health challenges such as Alzheimer’s disease, breast cancer, depression, heart disease, and epilepsy. Nonetheless, achieving this objective remains challenging, particularly in developing areas and regions with limited medical resources. The high incidence and growth rates of the aforementioned diseases further compound the challenges confronting the healthcare system in terms of diagnosis. This challenge primarily stems from two key factors: firstly, the low specialist-to-patient ratio, and secondly, the time-consuming and labor-intensive nature of the manual diagnosis, which heavily relies on specialized expertise. These issues often result in delayed treatment, exacerbating illness severity, and escalating medical costs. Consequently, there exists an urgent need for automated diagnostic approaches to address these pressing concerns.

AI-driven healthcare, emerging as a transformative force in the medical landscape, seeks to revolutionize clinical practices leveraging the capabilities of information technology. It represents a promising avenue for addressing critical disease diagnosis challenges in regions characterized by disparities in medical resources, garnering significant attention from both scholars and practitioners [1]. AI-driven healthcare entails the integration of medical data with intelligent technologies to enhance healthcare quality and productivity.

**Figure 1:**
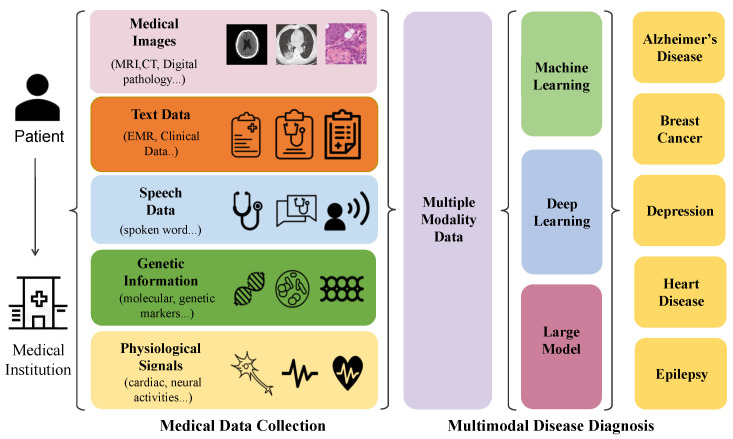
The diverse data types including images, speech, text, and genetic information can be produced in the clinical diagnostic process.

The clinical diagnostic process is inherently intricate, involving the generation and analysis of diverse data types encompassing images, speech, text, and genetic information (as depicted in Figure 1). This complexity stems from the synergistic interaction of multiple data sources, including images capturing anatomical structures, speech elucidating patient symptoms, textual descriptions of medical history, genetic information delineating inherent susceptibility, and physiological signals acquired through electrocardiograms (ECGs) and electroencephalograms (EEGs). Each modality furnishes unique and valuable insights that collectively contribute to a holistic understanding of patients’ physiological states.

- **Image.** Medical imaging tools such as computed tomography (CT), X-rays, magnetic resonance imaging (MRI), and digital pathology offer visual representations of internal structures and anomalies. These images serve as foundational components of a diagnosis, unveiling intricate details crucial for identifying and characterizing various medical conditions.
- **Text.** Textual data encompassing electronic health records, clinical notes, and medical literature constitute a narrative thread weaving through the patient’s medical journey, history, and contextual information vital for precise diagnosis.
- **Speech.** Speech recordings provide a unique avenue for understanding patients’ experiences and symptoms. This modality captures nuances such as tone, pace, and articulation, thereby adding a qualitative dimension to the diagnostic process.
- **Genetic data.** Genetic data introduce a molecular layer to elucidate inherent predispositions, susceptibilities, and genetic markers potentially influencing disease manifestation.
- **Physiological signals.** Signal data offer real-time snapshots of cardiac and neural activities. This dynamic modality effectively captures temporal variations, offering critical insights into abnormalities and patterns associated with cardiac or neurological diseases.

Numerous experts and scholars have actively participated in the collection and integration of medical data for diagnostic tasks, as evidenced by their contributions to various datasets [2,3,4,5,6]. Remarkably, these individuals not only curated and refined these datasets but also advocated for their accessibility and openness. For instance, the ADNI dataset, cited in references [7,8], has emerged as a cornerstone in neuroimaging and dementia research. This dataset incorporates diverse modalities such as structural and functional MRI, positron emission tomography (PET), and cerebrospinal fluid biomarkers, thereby offering a comprehensive perspective on disease progression. The availability of such datasets establishes a standardized framework for the development and evaluation of advanced diagnostic algorithms, particularly those leveraging machine learning and deep learning techniques. These methodologies play a pivotal role in extracting discernible features from multi-modal medical data and have witnessed significant advancements in recent years.

- **Machine learning approaches.** Machine learning methodologies have emerged as pivotal tools for medical diagnosis tasks, exemplified by techniques like Support Vector Machines (SVMs) [9] and Random Forests (RFs). SVMs excel in establishing optimal decision boundaries for classification, and are particularly adept at discerning intricate patterns within multidimensional data. On the other hand, RFs harness the strength of ensemble learning by amalgamating predictions from numerous decision trees, thereby enhancing model performance. The deployment of such machine learning techniques constitutes a substantial advancement in automated disease diagnosis, particularly in handling structured and well-defined datasets.
- **Deep learning models.** Deep learning models, as referenced in the literature [10,11,12], employ hierarchical neural networks to extract inherent patterns from medical data. For instance, Convolutional Neural Networks (CNNs) specialize in spatial feature extraction and prove beneficial in medical imaging applications, such as tumor detection in radiological scans. Conversely, Recurrent Neural Networks (RNNs) are well suited for sequence data analysis, enabling proficient performance in tasks like time series analysis or monitoring disease progression over time.
- **Large models.** Large models are designed to learn intricate feature representations from vast datasets [13,14,15,16,17,18]. In the field of medical data, large model approaches are expected to further improve the ability to capture and generalize complex features [19,20,21,22,23,24,25].

Existing reviews have offered insightful perspectives on research about automated disease diagnosis utilizing either machine learning or deep learning methodologies. However, these reviews predominantly concentrate on a singular modality or a single disease, whether focusing on a specific disease within multi-modal contexts, various disorders within a specific modality, or a single disease with exclusive reliance on a particular data type. In contrast, our review endeavors to explore the diverse modalities employed in the automatic diagnosis of distinct diseases. Although medical datasets generated by different disease diagnosis processes exhibit commonalities, distinct preferences for specific modalities prevail across different diseases. Consequently, this paper emphasizes general AI techniques applicable to different modalities and diseases, rather than solely focusing on a single disease or modality. Additionally, the latest advancements in large model-based specific disease diagnosis are introduced herein. To elucidate, we initially delineate available public datasets and the AI framework in automatic disease diagnosis, encompassing data pre-processing, feature engineering, model selection, and performance evaluation metrics. Subsequently, we expound upon reported works associated with various diseases. Lastly, a comprehensive discussion and outline of future avenues of exploration are presented to guide innovative solutions in this domain.

The remainder of this paper is structured as follows. In Section 2, we delve into the utilization of multi-modal data and AI in disease diagnosis, encompassing an exploration of public datasets and an overview of the overall processing framework. Section 3 provides a detailed exposition of the reported work, elucidating the methodologies, findings, and insights gleaned from recent research endeavors. In Section 4, we delineate the intricate challenges encountered in this field and outline potential avenues for future research and development. Finally, we encapsulate our findings and insights in the conclusion of this review in Section 5.

## 2. Multi-Modal and AI Used in Disease Diagnosis

Most diseases are typically only recognized by patients themselves after they manifest, and continuous data collection and monitoring can assist patients in achieving effective disease prevention. The advent of artificial intelligence has rendered the process of data accumulation more intelligent and efficient, thereby holding significant implications for disease prevention and control. This section elaborates on the comprehensive framework of artificial intelligence technology in medical diagnosis applications, encompassing data collection, model architecture construction, and model evaluation.

### 2.1. Datasets in AI-Based Disease Diagnosis Studies

Data collection plays a pivotal role in the development of machine learning models for disease diagnosis, serving as the bedrock upon which these models are constructed and trained. Many studies on AI-based disease diagnosis choose to utilize established open datasets to augment the research’s credibility and scope. In this section, we concentrate on the datasets employed in the research process across various diseases. For more detailed information on the data, please consult Table A1 in the Appendix A.

**Alzheimer’s disease.** The Alzheimer’s Disease Neuroimaging Initiative (ADNI) database [7,8], established in 2003, is widely recognized as one of the most prominent datasets for predicting AD. It encompasses various types of data, including brain imaging data such as MRI and PET scans, clinical data, biospecimen information, and genetic data. The patients in the ADNI database are categorized into different stages such as AD, MCI (Mild Cognitive Impairment), and NC (Normal Cognition). Another typical database is the longitudinal dataset called OASIS-3, which integrates multiple modalities [2], including neuroimaging, clinical biomarkers, and cognitive assessment. This dataset primarily investigates the progression of AD in 1378 individuals. Available at: http://www.oasis-database.org (accessed on 29 November 2023). Additionally, since 2006, the UK Biobank (UKB) [3,4,5] has amassed a substantial amount of data from participants, encompassing various fields such as environmental factors, lifestyle choices, sociodemographic information, overall health and well-being, as well as cognitive and physical assessments [6].

**Breast cancer.** The Cancer Genome Atlas (TCGA) [26] is a widely utilized dataset for predicting breast cancer. It involves MRI and CT scans, clinical records and genetic information. In the TCGA dataset, breast cancer is categorized into different subtypes, including Luminal A, Luminal B, HER2+, Basel, etc. The SAFHS [27] is a large-scale population-based natural language processing dataset developed by Harvard Medical School. Available at: http://www.ncbi.nlm.nih.gov/ (accessed on 29 November 2023). The Breast Ultrasound Images (BUSI) [28] was created in 2018 and contains normal, benign and malignant breast ultrasound images. Available at: https://scholar.cu.edu.eg/ (accessed on 29 November 2023). In the gene domain, Gene Expression Omnibus (GEO) [29] collects high-throughput functional genomics data for researchers, including microarrays, next-generation sequencing, and other forms. Available at: https://www.ncbi.nlm.nih.gov/geo/(accessed on 29 November 2023).

**Heart disease.** TLGS [30] is a long-term epidemiological research project for assessing the risk factors for cardiovascular diseases among residents of Tehran, Iran. Available at: https://endocrine.ac.ir/page/Tehran-Lipid-and-Glucose-Study-TLGS (accessed on 29 November 2023). In the text domain, the Acute Myocardial Infarction Dataset of the World Health Organization (WHO) collects from medical institutions and public health departments across various countries. Available at: http://www.who.int/ (accessed on 29 November 2023). It mainly studies the epidemiology, clinical characteristics, treatment methods, and prognosis of acute myocardial infarction and includes patient clinical information, diagnostic results, treatment measures, and other data. In the image domain, the Sunnybrook Cardiac Data (SCD) [31] dataset consists of 45 cine MRI images from different patients with various pathological conditions, including healthy individuals, hypertrophy, ischemic heart failure, and non-ischemic heart failure. Available at: https://www.cardiacatlas.org/sunnybrook-cardiac-data/ (accessed on 29 November 2023). In addition, the Automated Cardiac Diagnosis Challenge (ACDC) [32] database includes medical image data of normal subjects, ischaemic heart failure, dilated cardiomyopathy, hypertrophic cardiomyopathy, and right ventricular abnormalities. Available at: https://www.creatis.insa-lyon.fr/Challenge/acdc/ (accessed on 29 November 2023).

**Depression.** The Distress Analysis Interview Corpus-Wizard of OZ (DAIC-WOZ) [33] stands as one of the most popular speech datasets utilized for depression prediction. Available at: https://dcapswoz.ict.usc.edu/ (accessed on 29 November 2023). Its objective is to capture individuals’ verbal expressions of psychological distress and emotional stress through simulated interactions with AI. The corpus encompasses a broad spectrum of psychological disorders, including depression, anxiety, and post-traumatic stress disorder. Each entry within the dataset includes emotional annotations to furnish quantitative insights into the patient’s emotional state. The Multi-modal Open Dataset for Mental Disorder Analysis (MODMA) [34] is a multi-modal dataset tailored for mental disorders, featuring both clinically depressed patients and individuals from the normal population. Available at: http://modma.lzu.edu.cn/data/index/ (accessed on 29 November 2023). It comprises speech data and ECG data. Moreover, the Bipolar Disorder Corpus compiles textual data pertinent to bipolar disorder, aimed at facilitating researchers’ comprehension of the disorder’s characteristics, diagnosis, and treatment. The textual content within this repository encompasses diaries, medical records, clinical assessment reports, and other pertinent literature from individuals with bipolar disorder.

**Epilepsy.** The CHB-MIT [35] Database comprises EEG recordings collected from 22 pediatric subjects with intractable seizures and was established in 2010. Available at: http://physionet.org/ (accessed on 29 November 2023). The Bonn EEG time series database [36] involves EEG data obtained from a 128-channel acquisition system, featuring recordings from 5 patients identified as A, B, C, D, and E. Sets C and D encompass intracranial EEG recordings taken during seizure-free intervals, with set C recorded from within the seizure-generating area and set D from outside the seizure-generating area of epileptic patients. Available at: http://www.ukbonn.de/epileptologie/ag-lehnertz-downloads/ (accessed on 29 November 2023). Set E contains intracranial EEG data captured during epileptic seizures. Each set consists of 100 text files, each containing a single EEG time series represented in ASCII code and comprising 4097 samples. This database is devoid of artifacts, obviating the necessity for preprocessing prior to classifying the signals as healthy (non-epileptic) or unhealthy (epileptic). The Temple University EEG corpus database [37] represents an extensive collection of EEG data acquired between 2000 and 2013. Available at: http://isip.piconepress.com/projects/tuh$_$eeg/ (accessed on 29 November 2023). This repository encompasses diverse EEG clinical settings from approximately 10,874 patients. By incorporating a large cohort of patients and spanning a significant timeframe, the Temple University EEG corpus database affords opportunities for multifaceted analyses in EEG research. Researchers can exploit this invaluable repository to explore various facets of EEG data and advance the understanding of neurological conditions.

### 2.2. Framework for AI in Disease Diagnosis Modeling

**Figure 2:**
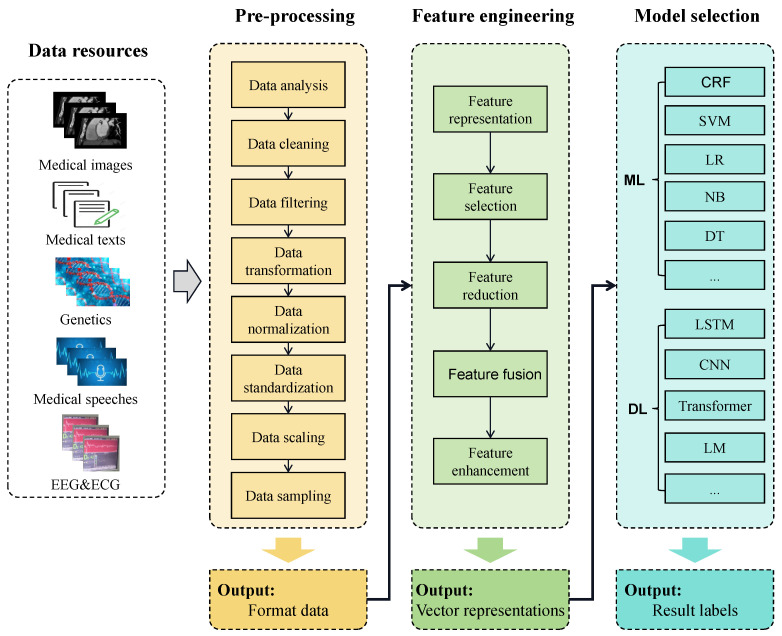
The framework for AI in disease diagnosis modeling (ML and DL denote machine learning and deep learning, respectively).

Up to now, AI models have been developed for a wide range of disease diagnoses. These models have undergone architecture designing and fine-tuning by leveraging diverse modalities of data such as medical images, medical texts, genetics, medical speeches, EEG, and ECG. Their applications span diagnostic classification, phenotype discovery, and other disease diagnosis tasks. In this section, we will focus on introducing well-known AI models and their intricate framework designs, including data preprocessing, feature engineering, and model selection (as shown in Figure 2).

#### 2.2.1. Pre-Processing

Pre-processing using machine learning and deep learning technologies is a crucial step for disease diagnosis. By preprocessing raw data, inaccurate or irrelevant information can be removed and key features relevant to disease diagnosis are extracted. Common preprocessing operations include data research and analysis, data cleaning, data filtering, data transformation, data normalization, data standardization, data scaling, data sampling, etc. Specifically:

**Data exploration.** It involves analyzing the number of samples, features, and their distributions of the dataset, which not only reveals the intrinsic properties of the dataset but also provides a solid foundation for the subsequent selection of preprocessing techniques.

**Data cleaning.** It aims to handle noisy or erroneous data, including removing duplicate entries, handling missing values, and correcting data errors or inconsistencies.

**Data filtering.** It is used to remove noise from a dataset, including low-pass filtering and high-pass filtering.

**Data transformation.** It involves converting raw data into different representations or forms.

**Data normalization.** It scales the data to a standard range or distribution, including min–max normalization, clipping normalization, standard deviation normalization, and z-score normalization.

**Data standardization.** Its primary function is to convert data from varying ranges and scales into a uniform standard format, such as FHIR HL7 [38], SNOMED CT [39] and DICOM [40], thus making data more suitable for machine learning and statistical analysis.

**Data scaling.** Data scaling enables data to map to specific ranges or intervals, ensuring comparability at different scales and effectively mitigating biases caused by scale differences.

**Data sampling.** The purpose of data sampling is to choose a subset of data from the primary dataset, thus forming a representative sample for analysis. In the case of imbalanced datasets, various sampling strategies can be utilized, including random sampling, stratified sampling, or oversampling/undersampling. These strategies can effectively address the issue of disparate class distributions in the dataset, ensuring accurate predictions for each class.

The above preprocessing operations aim to address issues such as noise, missing values, inconsistency, or specific data challenges. Facing different types of data (such as medical imaging, medical texts, genetic data, audio, and electrocardiogram signals), different preprocessing methods are usually required. Specifically:

**Medical imaging data.** Medical imaging data have a rich and complex spatial structure, consisting of a multidimensional matrix of pixels, each containing information about color and brightness. The preprocessing of medical imaging data mainly focuses on image resolution (number of pixels), color depth (color details in each pixel), and format (encoding methods such as portable network graphics (PNG)). For example, in the imaging process of medical images (such as X-rays, CT scans, and MRI), metal objects in the patient’s body (such as implants, dental restorations, surgical screws, etc.) and natural movements (appearing blurry or deformed in the image) can cause artifacts that affect the visualization of surrounding tissues. Metal artifact correction and motion correction are designed to handle such artifact situations. The imaging process is often susceptible to factors such as long or insufficient exposure time, scanning speed, radiation dose, and environmental interference, which can introduce random noise into the image. This requires the use of denoising methods such as wavelet denoising and median filtering. The lesions in medical images are often local abnormal changes, with some lesions having unclear boundaries and no clear boundaries with surrounding tissues. Data filtering operations such as smoothing filters and high-pass filters are needed to enhance the density, texture, and edge features of the image. In addition, images typically have various spatial resolutions, coordinate systems, and storage formats, so resampling techniques are needed to convert them to standard formats, such as from Medical Digital Imaging and Communications (DICOM) to PNG.

**Medical text data.** The first step in preprocessing medical text data is usually to decompose them into smaller units based on tokenization. During this process, special characters, punctuation, stopwords, and even spelling and morphological corrections will be removed to reduce data noise and redundancy. Additionally, because text data typically contain a large amount of vocabulary and semantic information, preprocessing typically considers factors such as word frequency, text length, and semantic association to reduce data dimensionality.

**Genetic data.** Genetic expression data usually include the expression levels of thousands of genes under different conditions or at different time points, complex and multidimensional. Also, gene expression data typically have a right-skewed distribution: most genes are concentrated at lower expression levels and a few genes have very high expression. Therefore, in preprocessing, apart from basic steps like data cleaning and normalization, logarithmic transformations (log), log base 10 (log10), square root transformations, etc., is required to convert the raw gene expression data into a form closer to a normal distribution.

**Medical speech data.** Original Speech data involve the target speaker’s voice and the other interference (e.g., background noise, voices of non-target speakers, reverberation, silence). Endpoint detection, pre-emphasis, framing, windowing, and other techniques are typically used to effectively suppress these interferences. Endpoint detection can detect silent segments in audio signals and segment audio sentences by threshold and short-term energy methods. Pre-emphasis technology is used to increase the importance of the high-frequency part for uniform information since important information in audio signals is often concentrated in the low-frequency part. Framing aims to slice the data to obtain short-term stable audio signals. Moreover, windowing effectively improves the issue of information leakage, with common window functions including the Hamming window, Hanning window, and rectangular window.

**EEG and ECG data.** Electroencephalogram (EEG) and electrocardiogram (ECG) signals are often interfered with by factors like blinking, movement of the body or electrodes, environmental noise, heartbeat fluctuations, power interference, or baseline drift. The preprocessing process is mainly to ensure signal purity. The Independent Component Analysis (ICA) technique is used to eliminate the interference from blinking and eye movement. Artifacts from cardiovascular and musculoskeletal system electrical activity can be removed using band-pass filters or the Discrete Wavelet Transform (DWT). Noise from power sources, harmonics, and movement of electrodes and wiring can be eliminated using filters of different frequencies.

#### 2.2.2. Feature Engineering

Feature engineering plays a crucial role in disease diagnosis using artificial intelligence technologies. It involves extracting, selecting, and transforming important information from original medical data to construct meaningful features for models. Specifically, feature engineering typically encompasses feature representation, feature selection, feature reduction, feature fusion, and feature enhancement.

**Feature representation.** Feature representation can transform raw input data into numerical representations that can be utilized by the model.

**Feature selection.** The redundant features can confuse machine learning models, while few features might not effectively and correctly classify data. Therefore, many researchers adopt feature selection techniques to choose appropriate features from extracted features. Common feature selection techniques include Information Gain, Chi-square Test, Mutual Information, Recursive Feature Elimination (RFE), Regularization, etc.

**Feature reduction.** When the number of extracted features is huge or they have not been properly normalized or scaled, feature reduction techniques are used to alleviate this problem. The most commonly used feature reduction technique is Principal Component Analysis (PCA), followed by other techniques such as Linear Discriminant Analysis (LDA), Sparse Encoding, and Factor Analysis.

**Feature fusion.** Feature fusion can enhance the efficiency of classifiers in detection tasks. It involves combining features extracted, selected, or reduced through different methods into a single set of parameters. This integration of features from various perspectives and methodologies offers a more comprehensive and in-depth understanding of the data. Typical feature fusion techniques include Topic Models, Multi-view Learning, and Knowledge Graph Fusion, among others.

**Feature enhancement.** Feature enhancement can enhance the representation of important features in data while weakening or eliminating the influence of irrelevant or noisy features. In disease diagnosis tasks, feature enhancement helps to more accurately distinguish different disease categories, thereby improving the accuracy and robustness of the model.

#### 2.2.3. Model Selection

According to the diagnostic methods of various diseases, artificial intelligence models are divided into two categories: traditional machine learning methods and deep learning methods.

In the era of rapid advancements in deep learning algorithms, traditional machine learning algorithms continue to be favored in the development of AI diagnostic models due to their unique advantages. They require fewer data points and offer better interpretability. However, traditional machine learning algorithms have clear drawbacks. They often require domain experts to pre-define the features to be learned before model training, resulting in additional manual costs and increased resource expenses. In the following sections, we will introduce commonly used machine learning methods in building AI diagnostic models.

**Conditional random fields (CRF).** CRF [41] has found numerous applications in disease diagnosis. It is a probabilistic graphical model that predicts labels by capturing contextual information of input sequences and considering the dependencies between adjacent labels in the sequence. In the context of disease diagnosis, the CRF model utilizes patient-specific input sequences (such as images, text, or genetic features) to model the conditional probability of the output sequence, representing different disease classifications or subtypes. This is achieved by defining feature functions and weights that represent the relationship between input and output sequences. Feature functions can include observation features (relating the current input to the output label) and transition features (relating the current output label to the previous output label).

**Support vector machine (SVM).** The SVM [9] is another commonly employed algorithm in disease diagnosis [42,43,44]. The SVM, introduced by Vapnik in 1990, operates on labeled data. It begins with extracting meaningful features from the input data (e.g., shape features, texture features, or local features for medical images; or disease-related features like biomarkers or keywords for biological signals or clinical text data). Then, leveraging the extracted features to train the SVM. The SVM seeks an optimal hyperplane that distinguishes different classes based on the position of input samples relative to the hyperplane in the feature space. Finally, disease diagnosis is derived from the predicted labels.

**Logistic regression (LR).** LR [45] maps the results of linear regression to the range (0, 1) using a logistic function, enabling the estimation of the probability of a sample belonging to a particular class. LR has been widely applied in disease diagnosis. It adjusts model parameters to maximize the likelihood function of the training data by learning the relationship between patient features (such as images, text, signals, or genes) and disease labels. Optimization algorithms like gradient descent are used to minimize the loss function and find the optimal model parameters.

**Naive Bayes (NB).** NB [46] is a probabilistic algorithm that does not rely on networks and performs well with high-dimensional features. In disease diagnosis tasks, the NB classifier learns the relationship between patient data features (such as medical images, clinical text, or biological signals) and disease labels, classifying patients into specific disease categories [47]. Furthermore, NB simplifies learning by independently classifying features within each class.

**Decision tree (DT).** The DT [48] is a commonly used data analysis algorithm [49]. It consists of terminal and non-terminal nodes, with each non-terminal node describing a condition or test for a data item. This technique is often employed in disease classification and is beneficial for association and regression tasks. Decision trees facilitate easy visualization and identification of various data aspects [1]. Numerous studies have utilized decision trees for disease diagnosis [50].

In addition to the aforementioned methods, many other typical traditional machine learning methods (e.g., K-means, RF, etc.) have been successfully applied to disease diagnosis tasks.

Unlike traditional machine learning approaches, deep learning methods can leverage all the information present in the data as features for training models, eliminating the need for predefined features. This significantly reduces the resource requirements associated with traditional machine-learning methods. Particularly in tasks such as AI diagnosis and prediction, deep learning methods demonstrate a compelling advantage over traditional machine learning methods, especially when abundant data are available. In the medical domain, where high precision is paramount, traditional machine learning methods are progressively being substituted by deep learning methods. The subsequent sections will highlight several widely used deep learning methods.

**Long short-term memory (LSTM).** LSTM [12], an improved version of the recurrent neural network (RNN), is composed of a series of fundamental units designed to address the issues of gradient vanishing and exploding in RNN through the use of gates and controlled features. Each unit includes an input gate, a cell state, a forget gate and an output gate. The input gate decides which feature information to update, while the forget gate is used to decide the amount of original feature information to discard. The cell state serves as a storage unit for feature information, and the output gate determines which feature information to output. Notably, LSTM excels in capturing contextual relationships and predicting subsequent data based on the preceding sequence. In the realm of disease diagnosis, LSTM finds utility in processing and modeling sequential data, including clinical texts and speech. Furthermore, LSTM has several variants, such as Bidirectional Long Short-Term Memory (Bi-LSTM) and Bidirectional Gated Recurrent Unit (BiGRU), which simultaneously predict the current state based on both the previous states and the future states.

**Convolutional neural networks (CNNs).** A CNN [10] possesses parallelism characteristics that LSTM does not have. Recently, the CNN has been widely applied in various medical imaging, laboratory reports, pathology reports, etc., and has achieved remarkable success in the field of AI-based diagnosis [51,52,53,54,55,56,57,58]. The concept of the “receptive field” in a CNN is essential as it decides the time frame for the CNN to make predictions based on contextual relationships. The window size and stride used in convolutions are parameters used to control the receptive field. In a CNN, a larger window size generates a larger receptive field, thus capturing more contextual relationships. However, this diminishes the influence of words closest to the prediction target in terms of their positional importance. Setting a larger stride in the CNN ignores certain contextual relationships while significantly increasing the overall computational speed.

**Transformer.** A transformer [11] is a deep learning model widely used for sequence-to-sequence tasks, having garnered significant acclaim in the field of natural language processing, particularly for machine translation, and subsequently finding broad research applications in other domains, including image processing. In the realm of medical diagnosis, A transformer proves valuable for processing and modeling diverse modalities of medical data, encompassing clinical texts, medical images, and time series data [59,60,61]. Primarily, leveraging the self-attention mechanism, the transformer computes relevance scores between each position in the input sequence and other positions. These scores facilitate weighted aggregation of input features, empowering each position to capture both global and local contextual information.

Moreover, to bolster modeling capabilities, the transformer introduces a multi-head attention mechanism, employing multiple self-attention sub-layers that focus on distinct facets of relevant information, effectively extracting features at varying levels and perspectives. Simultaneously, to retain positional information within the sequence, Transformer incorporates positional encoding, embedding positional details into the input representation, enabling the model to discern between different positions. Lastly, employing an encoder-decoder architecture, Transformer initially encodes the input sequence into high-dimensional representations, adeptly capturing the input data’s features, and subsequently, the decoder generates disease prediction outcomes based on the encoder’s output and target labels.

**Large model (LM).** With the emergence of foundational models [62,63], researchers have introduced a new paradigm that leverages deep learning methods, primarily relying on the emerging capabilities of large models (LMs) to handle more complex tasks through scale expansion. Unlike traditional specialized models trained for specific problems, a large universal foundational model only requires one training session to acquire a wide range of general knowledge and can subsequently adapt to various downstream tasks through prompts. This approach was initially introduced by language models as few-shot learners [64] and has gained widespread recognition with the introduction of groundbreaking models such as GPT-3.5 [13], GPT-4 [14], the LLaMA series (including LLaMA [15] and Llama2 [16]), PaLM [17], FLAN-T5 [65], and Alpaca [18].

Alongside technological advancements, large models targeting different data types, such as images (SAM [66]) and time series (TimeGPT-1 [67]), have also been developed, demonstrating their powerful performance. While these LMs have proven effective in various general domain tasks, they have yet to reach their full potential in specific medical domain tasks. In comparison to specialized models, LMs still exhibit certain gaps because specialized models are not only meticulously designed for specific tasks in terms of architecture but also guided by medical knowledge to better understand and capture subtle differences and semantic features in the data. In contrast, LMs currently fall short in this aspect. Consequently, there has been extensive research on LMs tailored for specific medical domains to better fulfill the requirements. XrayGPT [68] and XrayGLM serve as notable examples of large models applied in medical imaging. XrayGPT is an innovative conversational medical visual language model capable of analyzing and answering open-ended questions regarding chest X-rays. XrayGLM aims to become the first Chinese multi-modal medical LM proficient in interpreting chest X-ray images, showcasing remarkable potential in medical image diagnosis and multi-turn interactive dialogues. Available at: http://github.com/WangRongsheng/XrayGLM (accessed on 29 November 2023). Several LMs focused on medical text and speech have also emerged, including the Med-PaLM series (Med-PaLM [19] and PaLM 2 [20]), HuaTuo Algorithm [21], ChatDoctor [22], DoctorGLM [23], BianQue [24], and BioGPT [25], which have demonstrated significant potential in providing valuable assistance across various healthcare-related domains. In the realm of genetic data, Yang et al. [49] introduced GeneCompass, the first knowledge-based cross-species milestone foundational model, surpassing competitive state-of-the-art models in multiple tasks within a single species.

### 2.3. Performance Evaluation Metrics

In disease diagnosis tasks using artificial intelligence technology, performance evaluation metrics are commonly calculated based on the confusion matrix for binary classification tasks [69], which include four types of classifications: True Positive (TP), False Positive (FP), True Negative (TN), and False Negative (FN). As shown in Table 1, TP represents the correctly identified positive instances, i.e., the positive class correctly classified as positive. TN represents the correctly identified negative instances, i.e., the negative class correctly classified as negative. FP represents the falsely identified positive instances, i.e., instances of the negative class mistakenly classified as positive. FN represents the falsely identified negative instances, i.e., instances of the positive class mistakenly classified as negative. Total Positive refers to the sum of TP and FN, while Total Negative refers to the sum of TN and FP. True Classification is the sum of TP and FP, and False Classification is the sum of FN and TN. The definition of performance evaluation metrics is shown in Table 2.

In addition, other classification metrics such as the Area Under the ROC Curve (AUC-ROC) are also commonly adopted. The ROC curve plots the True Positive Rate (TPR) on the y-axis against the False Positive Rate (FPR) on the x-axis, where TPR = Recall(R) = TP/(TP + FN), FPR = FP/(FP + TN). The ROC curve illustrates the relationship among TPR and FPR at different classification thresholds. The AUC measures the area under the ROC curve, ranging from 0 to 1. An AUC of 1 indicates a model with perfect classification ability, while an AUC equals to 0.5 denotes that a model’s predictive performance is no better than random guessing.

## 3. Reported Works

### 3.1. Diagnosis of Alzheimer’s Disease

Alzheimer’s disease constitutes a progressive neurodegenerative disorder, characterized by cognitive decline, memory impairment, and compromised communicative abilities. In the realm of AI-driven diagnostic investigations for Alzheimer’s disease, medical imaging modalities such as MRI and PET are universally recognized as indispensable tools. They offer profound insights into the alterations of brain structure and functionality, thus furnishing critical information for diagnosis. Concurrently, the analysis of speech patterns has also surfaced as a promising domain. Changes in language and communication frequently serve as precursors to cognitive deterioration, making them significant markers for early detection. This section delves into and evaluates the pertinent literature on automated Alzheimer’s disease diagnosis, leveraging MRI, PET, speech, and other multi-modal strategies. A consolidated synopsis of the model and its attributes is presented herein, with detailed elaborations provided in Table 3.

**Magnetic resonance imaging (MRI).** MRI is pivotal in Alzheimer’s disease (AD) diagnostics, offering a non-invasive modality that provides intricate images capturing the brain’s structural and tissue details. There has been a substantial focus on harnessing morphological attributes from MRI scans as the central criterion for facilitating automated AD diagnosis. To illustrate, Li et al. [52] initiate the process by pinpointing the hippocampal regions in structural MRI (sMRI) images that are productive for diagnosis, drawing on prior knowledge. Subsequently, they deploy a deep learning architecture to distill distinctive patterns pertinent to AD diagnosis. Building upon this, Lian et al. [70] amalgamate a discriminative localization phase for brain atrophy with the subsequent stages of feature extraction and classification framework development. They introduce a Hierarchical Fully Convolutional Network (H-FCN) designed to autonomously and systematically discern patch-level and region-level indicative sites within the entire brain MRI scan. This model embraces a data-driven strategy that concurrently learns and amalgamates feature representations spanning multiple scales—from patch to region to subject level—to formulate a comprehensive AD diagnostic model. Addressing the nuances of brain atrophy, which pose significant diagnostic challenges in MRI imaging, Zhu et al. [59] unveil DA-MIDL, a novel deep learning framework endowed with a dual attention mechanism. This mechanism is adept at singling out the most salient pathological locales for AD diagnosis. DA-MIDL is composed of a patch network replete with spatial attention blocks, an attention Multiple Instance Learning (MIL) pooling module, and an attention-aware global classifier. The patch network is engineered to extract salient structural features from myriad local sMRI patches disseminated throughout the brain. The attention MIL pooling phase is adept at assigning variable weights to patch-level features, orchestrating them into a holistic representation of the entire brain’s architecture. This global representation forms the foundation for the subsequent AD diagnostic classifier.

Furthermore, the quantification of hippocampal volume attrition has been recognized as a seminal marker for AD diagnosis. Uysal et al. leverage semi-automatic segmentation software ITK-SNAP to calculate hippocampal volume metrics. They construct a dataset incorporating parameters such as age, gender, diagnostic status, and volumetric data for left and right hippocampal regions. Utilizing this dataset, they apply machine learning algorithms to effectively differentiate between Alzheimer’s disease (AD), Mild Cognitive Impairment (MCI), and cognitively normal (CN) cohorts.

**Positron emission tomography (PET).** While MRI images primarily yield extensive data on brain structure, they fall short of providing insights at the molecular level. This is where Positron Emission Tomography (PET) imaging gains its prominence. As a molecular imaging technique, PET scrutinizes specific biological processes such as protein aggregation, metabolic rates, or receptor concentrations using radiolabeled tracers. PET imaging thus offers an intricate depiction of biological and metabolic dynamics within the brain and is routinely employed in diagnosing and monitoring Alzheimer’s disease (AD). In the study by Chen et al. [60], a novel contrastive learning paradigm is introduced, utilizing brain 18F-FDG PET images to surmount the challenges associated with the paucity of data and the low signal-to-noise ratio, which are typical in PET images pertinent to AD prediction. They implement a data augmentation strategy to amplify the volume of training data, and they apply the adversarial loss to expand the distances between features of different classes while consolidating the similarities within the same class.

Furthermore, they develop a dual convolutional mixed attention module, fine-tuning the network’s proficiency in discerning diverse perceptual fields. By aligning the predictive outcomes of individual PET slices with clinical neuropsychological evaluations, they advance a diagnostic methodology conducive to refining AD diagnoses. Baydargil et al. [71] deliver an unsupervised adversarial parallel model tailored for the anomaly analysis in AD, sharply delineating AD, mild cognitive impairment (MCI), and normal control groups. The model exhibits robust classification with rates and area under the curve (AUC) scores reaching 96.03% and 75.21%, respectively, underscoring its effective discriminative performance. Lu et al. lay the groundwork for a cutting-edge deep learning infrastructure, utilizing FDG-PET metabolic imaging to pinpoint subjects with symptomatic pre-AD in the MCI phase, setting them apart from other MCI cohorts (non-AD/non-progressive). They pioneer a multi-scale deep neural network that reports a classification precision of 82.51%, relying solely on a single-modal metric (FDG-PET metabolic data). Cheng et al. [53] present an innovative classification scheme that amalgamates a two-dimensional Convolutional Neural Network (CNN) with a Recurrent Neural Network (RNN). Their strategy is oriented towards deconstructing 3D images into a succession of 2D slices to capture the features inherent to 3D PET imagery. Within this framework, they architect a hierarchical 2D cellular neural network tasked with the extraction of intra-slice features, while the Gated Recurrent Unit (GRU) within the RNN is deployed to elucidate inter-slice features that contribute to the final classification outcome.

**Speech.** The manifestation of Alzheimer’s disease (AD) in speech signals offers a distinctive avenue for diagnosis, as individuals with AD exhibit notable speech pattern alterations compared to those without the condition. Employing speech recognition technology for AD diagnostics is not only non-invasive and safe but also cost efficient, making it an appealing methodology for widespread application. Before the infusion of deep learning into the field, traditional approaches to speech analysis for AD diagnosis relied heavily on manual feature extraction. Techniques such as analysis of static features, utilization of feature sets like ComParE 2016 and eGeMAPS, as well as Mel-Frequency Cepstral Coefficients (MFCC), were common practices. These extracted features were then analyzed using machine learning classifiers, including logistic regression, random forests, and support vector machines, to distinguish between affected and healthy individuals. Studies by Hason et al. [72], Hernández et al. [73], and Yu et al. [74] are examples of such research efforts.

With the advent of deep learning, there has been a paradigm shift in research methodologies for AD diagnosis. Deep learning techniques have taken precedence, given their ability to automatically extract complex patterns from raw data without the need for manual feature selection. In this context, Lopez et al. [55] have made strides in early AD detection by implementing classical Multilayer Perceptrons (MLPs) and Convolutional Neural Networks (CNNs), illustrating the potential of deep learning in enhancing diagnostic accuracy. Further advancing the field, Liu et al. [75] leveraged an Automatic Speech Recognition (ASR) model to derive speaker-independent bottleneck features, which are highly discriminative and robust. They coupled this with a CNN for modeling local context and an RNN for capturing the global context within speech. An attention mechanism was integrated to selectively focus on the most salient features for AD detection, improving the model’s interpretability and effectiveness. Additionally, Bertini et al. [76] introduced an end-to-end model for AD detection, innovatively applying SpecAugment [77] for data augmentation to enhance the robustness and generalizability of the model against variability in speech data. They then utilized the auDeep [78] autoencoder, followed by fully connected layers for feature learning and classification, streamlining the process from raw speech input to the diagnostic output. This end-to-end approach simplifies the pipeline and potentially improves the model’s accuracy and applicability in clinical settings.

**MRI-PET image fusion.** The integration of MRI and PET imaging modalities has yielded a synergistic approach in medical diagnostics, particularly for disorders such as Alzheimer’s disease (AD). This technique of image fusion leverages the unique strengths of each imaging method to offer a more holistic representation of the brain’s structure and function. The pioneering work of Shi et al. [79] introduced the multi-modal Stacked Denoising Predictive Network (MM-SDPN). This algorithm is structured in two phases specifically tailored to merge and learn from the feature representations of multi-modal neuroimaging data. This integration enhances the diagnostic process for Alzheimer’s disease, offering a deepened insight into the complex interactions between different types of brain changes associated with the disease. Sharma et al. [80] took a different approach, utilizing wavelet packet transform as their method of fusing MRI and PET images. Their methodology involves an eight-layer Convolutional Neural Network (CNN) that meticulously extracts features across multiple layers. The extracted features are then processed through an ensemble of non-iterative Random Vector Functional Link (RVFL) networks. This ensemble strategy aims to robustly capture the intricate patterns from the fused data for accurate AD diagnosis.

Further advancing the field, Zhou et al. [81] proposed a unique method for latent representation learning that encompasses data from various modalities, including MRI, PET, and genetic information. Their approach focuses on deducing latent representations and then projects these representations into the label space for diagnostic purposes. This technique underscores the potential of combining structural, functional, and biological data to enhance the accuracy of Alzheimer’s disease diagnostics. Addressing the potential issue of overfitting when dealing with the fusion of high-dimensional data, Ning et al. [72] developed a relation-induced multi-modal shared representation learning approach. Their model is an integrative framework that combines the processes of representation learning, dimensionality reduction, and classifier design. It operates by learning bidirectional mappings between the original feature space and a shared representation space, thereby distilling the essence of multi-modal inputs into a cohesive, shared format that is conducive to diagnostic analysis. These studies illustrate a growing trend in leveraging sophisticated computational models and algorithms to enhance the accuracy and reliability of Alzheimer’s disease diagnostics by capitalizing on complementary information from multiple imaging modalities.

**Speech–Text fusion.** The nuanced extraction of acoustic features from speech datasets, coupled with the semantic analysis of textual data, fosters an enriched comprehension of Alzheimer’s disease (AD). By amalgamating speech and text data, a more extensive spectrum of AD-related features is captured, bolstering the diagnostic accuracy for this condition. Historically, the nascent stages of AD research leveraged machine learning techniques for analytical purposes. Shah et al. [42] focused on the extraction of word-level duration features, datasets on pause rates, and measures of speech clarity. They explored a variety of models, such as logistic regression, random forest, support vector machine (SVM), extreme gradient boosting, and neural networks in isolation and in combination, targeting both classification and regression tasks. Martinc et al. [43] commenced with spectrum subtraction for noise abatement, progressing to the use of a bag-of-n-grams approach for textual feature extraction. Concurrently, they extracted eGeMAPS features from speech data. A suite of classifiers, including XGBoost, SVM, random forest, logistic regression, and linear discriminant classifiers, was then deployed for classification tasks.

In the landscape of recent advancements, deep learning techniques have increasingly been harnessed for the automated diagnosis of Alzheimer’s disease. Cai et al. [82] applied Graph Neural Networks (GNNs) for the extraction of textual features and introduced audio data by utilizing the WavLM model to extract salient audio features. They then integrated these features with text features via various methodologies. Mei et al. [83] extracted a plethora of features comprising static acoustic features, the ComParE 2016 feature set, the eGeMAPS feature set, along with feature vectors from the wav2vec2 pre-trained model, and the Hubert pre-trained model for AD detection. They meticulously fine-tuned the wav2vec2.0 model on speech from assorted frequency bands, culminating in a remarkable accuracy of 87% and an RSME of 3.727. Agbavor et al. [84] procured deep representation features through data2vec and wav2vec2, subsequently refining an end-to-end model with fully connected layers for enhanced AD detection efficacy.

**Other models.** A diverse array of molecular and multi-omics approaches, including RNA-seq, single nucleotide polymorphisms (SNPs), protein sequences, and integrated omics data, have been employed to unravel the complexities of Alzheimer’s disease diagnosis. For instance, groundbreaking work by Li et al. [84], Taeho et al. [85], Xu et al. [86], Javier et al. [87], and Park et al. [88] has significantly contributed to the field by leveraging these techniques. Further, Park et al. [88] have pioneered a deep learning approach tailored for AD prediction that synergistically utilizes multiple heterogeneous omics data. In a similar vein, Golovanevsky et al. [89] have devised a multi-modal Alzheimer’s Disease Diagnostic framework (MADDi), ingeniously combining neural networks with attention mechanisms to harness the power of imaging, genetic, and clinical data for enhanced AD diagnostic precision. In addition to these genomic and proteomic strategies, electrophysiological methods such as EEG have been instrumental in AD diagnosis. Notable research by Djemili et al. [90], Pandya et al. [91], Kim et al. [92], along with studies cited as [93], have demonstrated the utility of EEG in capturing the neurophysiological hallmarks of Alzheimer’s disease, adding a valuable dimension to the diagnostic toolkit.

**Table 3:** Summary of different medical features for Alzheimer’s disease diagnosis.

Literature	Feature Name	Modality	Dateset	Results
Li et al. [52]	Hippocampal morphology feature	MRI	ADNI	0.939 (AUC)
Lian et al. [70]	Original MRI scan feature	MRI	ADNI	0.9 (ACC); 0.95 (AUC:AD vs. NC)
Zhu et al. [59]	Patch proposals selected from the MRI scans	MRI	ADNI, AIBL	0.9193 (ACC: AD vs. NC vs. MCI) 0.9287 (AUC)
Chen et al. [60]	optimized anchor data from brain 18F-FDG PET slices	PET	ADNI	0.9193 (ACC: AD vs. NC vs. MCI) 0.9287 (AUC)
Baydargil et al. [71]	Original PET slices	PET	ADNI	0.9603 (ACC: AD vs. NC vs. MCI) 0.7521 (AUC)
Cheng et al. [53]	a sequence of 2D slice groups from 3D PET	PET	ADNI	0.9528 (AUC: AD vs. NC)
Shi et al. [79]	high-level features of MRI and PET	MRI, PET	ADNI	0.9713 ± 0.0444 (ACC: AD vs. NC)
Sharma et al. [80]	Fused image by wavelet packet transform (WPT)	MRI, PET	ADNI	0.9603 (ACC: AD vs. NC vs. MCI) 0.7521 (AUC)
Zhou et al. [81]	magnetic resonance imaging (MRI), positron emission tomography (PET), and genetic data	MRI, PET, Gene	ADNI	-
Ning et al. [72]	magnetic resonance imaging (MRI) and positron emission tomography (PET)	MRI, PET	ADNI	0.976 (AUC: AD vs. NC) 0.969 (ACC: AD vs. NC)
Li et al. [84]	RNA-seq	Gene-based	GEO	0.859 (AUC), 0.781 (ACC)
Taeho et al. [85]	SNP	Gene-based	ADNI	0.82 (AAUC)
Xu et al. [86]	protein	sequence Gene-based	UniProt	0.857 (ACC)
Javier et al. [87]	genetic variation data	Gene-based	ADNI	0.719 (ACC)
Park et al. [88]	Multi-omics data	Gene-based	GEO	0.823 (ACC)
Golovanevsky et al. [89]	imaging, genetic, and clinical data	Gene-based	GEO	0.9688 (ACC)
Djemili et al. [90]	statistical characteristics (1. Maximum value in each IMF. 2. Minimum value in each IMF. Mean of the absolute values in each IMF. 4. Standard deviation in each IMF.)	EEG	Bonn dataset	The classification accuracy for normal and abrupt cessation electroencephalogram (EEG) signals is 1, while the classification accuracy for intermittent and abrupt cessation EEG signals reaches 0.977
Pandya et al. [91]	Amplitude, period and waveform offset of K-Complex	EEG	Private dataset	-
Kim et al. [92]	EEG segment with respect to RP(Absolute power of EEG signals in three different frequency bands)	EEG	Private dataset	0.75 (ACC)
Deepthi et al. [93]	Frequency domain features extracted by Fast Fourier Transform (FFT)	EEG	ADNI	-
Hason et al. [72]	MFCC	speech	ADReSS	Accuracy: 0.822
Hernández et al. [73]	Speech duration, descriptive statistical variables	specch	private dataset	Accuracy: 0.8
Yu et al. [74]	Based on phoneme characteristics, pronunciation coordination characteristics, and pitch variance	speech	private dataset	Accuracy: 0.93
Lopez et al. [55]	Linear features include spectral domain features and time domain features, such as harmonicity, spectrum centroid, formants, etc. Nonlinear characteristics include fractal dimension, permutation entropy, multi-scale permutation entropy, etc.	speech	private dataset	Accuracy: 0.89
Liu et al. [75]	Bottleneck feature vector (depth representation feature)	speech	Dementia- Bank Pitt	F1: 0.7802
Bertini et al. [76]	spectrogram	specch	Dementia- Bank Pitt	Accuracy is 0.933, F1 score is 0.885
Shah et al. [42]	Word-level duration feature set, pause rate data set, speech intelligibility feature set	speech, text	ADReSS-M	Accuracy: 0.696, RMSE: 4.8
Martinc et al. [43]	bag-of-n-grams features (text) eGeMAPS feature set (voice)	speech, text	Dementia- Bank Pit	Accuracy: 0.9167
Cai et al. [82]	GNN (text features) WavLM (voice features)	Speech, text	Dementia- Bank Pit	Accuracy: 0.8484 ± 0.0544
Mei et al. [83]	Silent characteristics ComParE 2016 feature set, eGeMAPS feature set wav2vec2 pre-trained model feature vector Hubert pre-trained model feature vector	Speech, text	AADReSS-M	Accuracy: 0.87, RMSE: 3.727
Agbavor et al. [84]	data2vec, wav2vec2	Speech, text	ADReSSo	F1: 0.728, RMSE: 3.493

### 3.2. Diagnosis of Breast Cancer

Breast cancer, originating in the breast cell tissue, stands as a pivotal health challenge for individuals across the globe. The key to enhancing survival and ensuring a better quality of life for those impacted by this disease lies in early detection and an integrated approach to treatment, involving a diverse team of medical professionals. The conventional diagnostic toolkit for breast cancer includes mammography, which is instrumental in visualizing breast tissue and identifying any irregularities that may indicate the presence of cancerous cells. Clinical breast exams conducted by healthcare professionals also play a significant role in early detection, as they involve a thorough palpation of the breast tissue to detect lumps or other changes. Additionally, gene screening is becoming increasingly important in breast cancer diagnosis, particularly for women with a family history of the disease, as it can identify inherited genetic mutations that may elevate the risk of breast cancer, such as mutations in the BRCA1 and BRCA2 genes. In this section, the diagnostic methodologies driven by the aforementioned modalities are rigorously explored and demonstrated. To provide a clear and concise representation of the various models and their attributes, reference is made to the details encapsulated in the accompanying tables, labeled as Table 4. These tables present a summarized outlook of the models, delineating their features, performance metrics, and other pertinent details that contribute to the overarching domain of breast cancer diagnosis.

**X-ray mammography.** Breast Lesion Classification is a critical facet of breast cancer diagnosis, as it aims to accurately differentiate between benign and malignant lesions discovered during screenings. X-ray mammography remains the cornerstone of early breast cancer detection, enabling physicians to spot minuscule masses or calcifications that could indicate the presence of cancer cells within the breast tissue. To augment the diagnostic efficiency for breast lesions, Al-antari et al. [94] have presented a comprehensive Computer-Aided Diagnosis (CAD) system that harnesses the power of deep learning, leveraging data from the DDSM and INbreast databases, which are prominent digital mammography datasets. The innovation began with the utilization of a You Only Look Once (YOLO) [95] deep learning detector specifically calibrated for the identification of breast lesions across whole mammograms. Subsequently, Al-antari et al. assessed and fine-tuned three deep learning classifiers—the standard feedforward CNN, ResNet-50, and InceptionResNet-V2—for the nuanced task of breast lesion classification.

Furthering the advancement in this domain, Yeman et al. [96] introduced an inventive approach employing a parallel deep Convolutional Neural Network (CNN) designed to analyze and learn from the symmetrical deep features extracted from the bilateral views of breast X-ray images. They innovatively computed the probability of pixels being part of a lesion by examining the local line and gradient direction features distribution, which then pinpointed the centers of suspected lesions. A global threshold was applied to these likelihood images to discern potential lesion-bearing regions. Ensuring symmetry, right and left breast X-ray images were horizontally flipped for congruent orientation, and the analysis proceeded with patched images fed into two mirrored deep CNN structures. The concatenated deep features from this twin-CNN setup were introduced into a Neural Network (NN) classifier, which achieved a remarkable prediction accuracy rate of 93.33%. In another groundbreaking work, Riyadh et al. [97] conceived a novel mixed deep learning Computer-Aided Diagnosis system for breast lesions, which combined a backbone residual deep learning network to generate profound features with a transformer that incorporates self-attention mechanisms for the classification of cancer. This innovative model achieved a perfect 100% accuracy rate for binary classification and an impressive 95.80% for multi-class prediction tasks, a testament to the potential of mixed AI models in discerning between benign and malignant breast tissues with high precision.

**Magnetic resonance imaging.** Breast MRI is a powerful diagnostic tool that excels in providing detailed insights into breast cancer lesions, surpassing other imaging modalities in delivering precise evaluations of lesion size, location, and type. The robust magnetic field and non-ionizing radiation technique of MRI make it a choice modality for comprehensive breast cancer assessment. Abunasser et al. [98] have made significant strides in the realm of breast MRI by training six advanced deep learning models, each with the capability to classify eight specific types of breast cancer, encompassing both benign and malignant forms. Their study incorporated a diverse set of models including their own proposed Breast Cancer Neural Network (BCNN), as well as Xception, InceptionV3, VGG16, MobileNet, and ResNet50, all fine-tuned to analyze MRI images for this purpose. These models demonstrated remarkable accuracy in their classification tasks, with rates of 97.54%, 95.33%, 98.14%, 97.67%, 93.98%, and 98.28% respectively, showcasing their potential to serve as reliable diagnostic aides. Complementing these efforts, Huang et al. [99] embarked on a comprehensive study involving the extraction of an extensive array of 4198 radiomic features from pre-biopsy multiparametric MRI datasets, which included dynamic contrast-enhanced T1-weighted images, fat-suppressed T2-weighted images, and apparent diffusion coefficient maps. In their pursuit of optimal feature selection, they employed a suite of methodologies such as the Least Absolute Shrinkage and Selection Operator (LASSO), Recursive Feature Elimination (RFE), Maximum Relevance Minimum Redundancy (mRMR), Boruta, and Pearson correlation analysis. Leveraging these strategically chosen features, Huang et al. proceeded to construct 120 diagnostic models that varied by classification algorithms, MRI sequence-segmented feature sets, and the employed selection strategies. These models were adeptly designed to not just categorize breast cancer lesions but also to predict cancer molecular subtypes and androgen receptor expression, potentially offering a nuanced approach to personalized cancer care.

**Ultrasound images.** The field of medical imaging for breast cancer diagnosis has been greatly enhanced by the incorporation of artificial intelligence, with ultrasound imaging being a key focus due to its safety and non-invasive nature. Jabeen et al. [100] introduced a cutting-edge classification framework specifically designed for ultrasound images, which effectively combines the prowess of deep learning with optimal feature selection techniques. This framework is composed of a structured five-step process: (i) Data augmentation is applied to expand the dataset, thereby providing a more robust foundation for training Convolutional Neural Network (CNN) models. (ii) The pre-trained DarkNet-53 model is adapted by modifying its output layer to align with the categories of the augmented dataset. (iii) Transfer learning is employed to train this modified model, with feature extraction carried out from the global average pooling layer. (iv) Two enhanced optimization algorithms, the Improved Differential Evaluation (RDE) and Improved Grey Wolf (RGW), are utilized for the selection of the most discriminative features. (v) A novel, probability-based sequential method is used to combine these optimally selected features, followed by the application of machine learning algorithms for the final classification task. The implementation of this framework on the Augmented Breast Ultrasound Images (BUSI) dataset resulted in an impressive highest accuracy of 99.1%, demonstrating its potential to significantly improve diagnostic processes.

Building on the momentum of innovation in the field, Ragab et al. [101] spearheaded the development of an Integrated Deep Learning Clinical Decision Support System for Breast Cancer Diagnosis and Classification (EDLCDS-BCDC). This innovative technology is engineered to detect the presence of cancer through the analysis of ultrasound images. The process involves an initial preprocessing stage using Wiener filtering and contrast enhancement to prepare the images. Image segmentation is then carried out using the Chaos Krill Herd Algorithm (CKHA) and Kapur Entropy (KE). The feature extraction is performed through an ensemble of three sophisticated deep-learning models, namely VGG-16, VGG-19, and SqueezeNet. The final stage of the classification process employs the Cat Swarm Optimization (CSO) algorithm to optimize a Multi-Layer Perceptron (MLP) model, ensuring precise categorization of the cancer images. Both these studies showcase the innovative intersection of deep learning and optimization algorithms in improving the accuracy and efficiency of breast cancer classification using ultrasound imaging.

**Medical text data.** The use of advanced natural language processing (NLP) techniques to analyze and classify medical data, including patient self-reports and medical records, has become increasingly prevalent in breast cancer research. Leveraging the power of these techniques can provide valuable insights and assist in the early detection and treatment of breast cancer. Kumar et al. [102] tailored a BERT-based model to specifically address the classification of breast cancer-related posts on Twitter, as described in Shared Task 8 of SMM4H-2021. Their approach was to employ BlueBERT [103], which is pre-trained on a comprehensive biomedical corpus acquired from PubMed, enhancing the model’s understanding of medical terminology and context. To bolster the model’s resilience against adversarial inputs, they incorporated gradient-based adversarial training, which ultimately resulted in the model achieving F1 scores of 0.8625 on the development set and 0.8501 on the test set, reflecting high accuracy in the automatic classification of breast cancer mentions in social media posts.

Further innovations in NLP, as seen in the works of Chen et al. [104] and Zhou et al. [105], push the boundaries of model interpretability and domain-specific accuracy. Chen et al. [104] took the capabilities of BERT further by integrating semantic trees into the model, thus constructing an interpretable neural network. They harnessed a capsule network with multiple attention heads to refine the semantic representations, while backpropagation and dynamic routing algorithms were implemented to provide local interpretability. This level of interpretability is particularly important in medical applications where understanding the reasoning behind a model’s prediction is as crucial as the prediction itself. Zhou et al. [105] explored the benefits of pre-training BERT on a cancer-specific dataset, which aimed to enhance the model’s ability to extract breast cancer phenotypes from pathology reports and clinical records. Their findings underscore the significance of domain-specific pre-training, as it substantially improved the performance of the model, making it more attuned to the nuances of cancer-related data. Addtionally, Deng et al. [106] investigated the potential assistance provided by advanced language models like GPT-4 in the context of breast cancer diagnosis. The authors emphasized GPT-4’s capability to rapidly mine crucial information from extensive medical records, which could potentially influence the diagnosis of breast cancer. By automating the extraction of key data points, GPT-4 could enhance the accuracy and efficiency of diagnostic procedures, supporting healthcare professionals in making informed decisions. These studies collectively highlight the transformative impact that state-of-the-art NLP models can have on the medical field, particularly in the realm of breast cancer diagnosis and classification.

**Genetic data.** Human cancer is a heterogeneous disease caused by stochastic cellular mutations and driven by various genomic alterations [107,108]. Currently, numerous research efforts are focused on utilizing genetic data and artificial intelligence algorithms to develop diagnostic models to enhance the clinical efficiency and accuracy of breast cancer diagnosis [109,110,111]. Presently, artificial intelligence techniques in breast cancer diagnosis research based on genomics primarily focus on RNA-seq data, single nucleotide polymorphisms (SNPs), protein sequences, and the integration of multi-omics data. (1) RNA-seq. Xu et al. [112] proposed a multi-granularity cascade forest (gcForest) for predicting four subtypes of breast cancer (Basal, Her2, Luminal A, and Luminal B). They compared the gcForest classifier with three different machine learning methods (KNN, SVM, and MLP). The results showed that gcForest showed a higher accuracy score of 92%. (2) MicroRNA. Sherafatian et al. [50] employed three tree-based algorithms (Random Forest, Rpart, and tree bag) to classify breast cancer subtypes (Luminal, HER2-enriched, basal) using miRNA data from TCGA. The results showed that Rpart achieved the best classification performance. For the Luminal subtype, the accuracy, sensitivity, and specificity were 88.9%, 82.4%, and 95.4%, respectively. For the HER2-enriched subtype, the accuracy, sensitivity, and specificity were 90.2%, 93.9%, and 86.4%, respectively. For the basal subtype, the accuracy, sensitivity, and specificity were 84.5%, 75%, and 94%, respectively. (3) Multi-omics data. Mohaiminul et al. [58] proposed a comprehensive deep-learning framework for classifying molecular subtypes of breast cancer. The framework utilized copy number alteration and gene expression data from the METABRIC. The results achieved an accuracy of 76.7% and an AUC of 83.8%.

**Table 4:** Summary of different medical features for breast cancer diagnosis.

Literature	Feature Name	Modality	Dateset	Results
Al-Antari et al. [94]	Original X-ray mammographic data	X-ray	CBIS-DDSM and DDSM	0.985 (ACC)
Yeman et al. [96]	Breast lesion detection from entire mammograms by object detection model	X-ray	DDSM and INbreast	ACC of three models: 94.50%, 95.83%, and 97.50%
Riyadh et al. [97]	Extracted patches centered on the points from the original X-ray	X-ray	General Electric, Siemens, and Hologic	0.933 (AUC)
Abunasser et al. [98]	Original MRI data	MRI	Kaggle depository	98.28 (F1-score)
Huang et al. [99]	multi-parametric MRI	MRI	Private dataset	Multilayer Perceptron (MLP): 0.907 (AUC) and 85.8% (ACC)
Jabeen et al. [100]	Original ultrasound images data	Ultrasound Images	BUSI dataset	99.1% (ACC)
Ragab et al. [101]	Segmented regions from original	ultrasound images Ultrasound Images	-	96.92% (ACC)
Kumar et al. [102], Peng et al. [103]	Word embedding	Text	witter self-report	F1: 0.8501
Chen et al. [104]	Word embedding, syntactic structure	Text	Shanghai Ruijin Hospital Molybdenum Mammography X-ray Report	Mi-P(%) = 91.58 Mi-R(%) = 91.58 Mi-F1(%) = 91.58 Ma-P(%) = 75.95 Ma-R(%) = 79.73 Ma-F1(%) = 77.14
Zhou et al. [105]	mutil feature	Text	private dataset	exact match and lenient match, macro-F1: 0.876, 0.904
Xu et al. [112]	RNA-seq	Gene-based	Medical Records	-
Sherafatian et al. [50]	miRNA	Gene-based	TCGA	92% (ACC)
Mohaiminul Islam M et al. [58]	Copy number alteration (CNA), RNA-seq	Gene-based	METABRIC	76.7% (ACC), 83.8% (AUC)
Sun et al. [108]	Clinical, CNV, RNA-seq	Gene-based	METABRIC	82% (AUC)

### 3.3. Diagnosis of Depression

Depression is a common mental health disorder characterized by persistent feelings of sadness, hopelessness, and a lack of interest or pleasure in daily activities. It can affect a person’s thoughts, emotions, and physical well-being, often leading to challenges in daily functioning. Depression varies in severity, and its impact on individuals can range from mild to severe. In the realm of diagnosis, text, speech, and EEG analysis have emerged as crucial tools for assessing and understanding depression. These modalities offer valuable insights into an individual’s mental state, providing a nuanced understanding of their emotional well-being. This section aims to delve into various approaches and methodologies related to the diagnosis of depression using these modalities. This section provides a summarized overview of the model and its features, as detailed in the accompanying Table 5.

**Medical text data.** Aragon et al. [58] introduced a sophisticated deep emotional attention model tailored for the detection of anorexia and depression. This model integrates nuanced sub-emotion embeddings with the advanced architectures of Convolutional Neural Networks (CNNs), Gated Recurrent Units (GRUs), and attention mechanisms to attain high predictive accuracy. Verma et al. [113] explored depression detection through the analysis of tweet data, utilizing four established machine learning models: Naive Bayes, Support Vector Machines (SVMs), K-Nearest Neighbors (KNNs), and Random Forest. Of these, the Random Forest model demonstrated superior performance, achieving an impressive accuracy peak of 78%.

Furthering the field, Ghosh et al. [114] adopted a novel deep multi-task learning strategy that simultaneously addresses emotion recognition and depression detection. Their findings suggest that the multi-tasking framework significantly boosts the efficacy of both tasks when learned concurrently. Xu et al. [115] ventured into the domain of psychological health with the introduction of their Linguistic Landscape Model (LLM). This model was rigorously tested across a spectrum of tasks, including psychological stress classification, depression severity assessment, suicide ideation detection, and suicide risk evaluation. The empirical results underscored the LLM’s robust performance, placing it on par with the leading task-specific models in the field. Lastly, Qi et al. [116] presented an all-encompassing benchmark that capitalizes on supervised learning techniques alongside the LLM framework, with a specific emphasis on the capabilities of the GPT series. Their research offers an in-depth analysis of these advanced LLMs, particularly in their application to cognitive distortion diagnosis and suicide risk stratification. This study not only highlights the models’ proficiency in capturing and interpreting complex emotional states but also provides a critical examination of their inherent potential and current limitations within the psychological domain.

**Speech.** From the initial forays into the realm of machine learning for depression diagnosis, a vast array of approaches has emerged. Liu et al. [117] introduced a multi-task ensemble learning technique that utilizes speaker embeddings to facilitate depression classification. Long et al. [118] devised an innovative multi-classifier system dedicated to depression recognition, distinguished by its synthesis of various speech types and emotional nuances. Jiang et al. [119] developed the Ensemble Logistic Regression Model for Depression Detection (ELRDD), representing a significant stride in predictive modeling. Complementing this, Liu et al. [120] proposed an inventive decision tree-based method for the fusion of speech segments, aimed at bolstering the accuracy of depression recognition.

As deep learning forges ahead, its methodologies are increasingly being adopted for diagnosing depression. Yin et al. [121] presented a deep learning model that harnesses the strengths of parallel Convolutional Neural Networks (CNNs) and Transformers, balancing effective information extraction with computational tractability for depression detection. Adding to this body of work, Tasnim et al. [122] examined the predictive utility of two acoustic feature sets—conventional handcrafted features and those derived from deep representations—in assessing depression severity through speech analysis. He et al. [123] proposed a hybrid approach combining handcrafted elements with deep learning features to precisely gauge depression severity from speech. Dubagunta et al. [124] conducted an exploration into methods for modeling speech source-related information in the context of depression, mindful of the potential neural physiological changes impacting vocal cord function. Zhao et al. [125] sought to advance depression detection by tapping into inherent speech information, advocating for a Long Short-Term Memory (LSTM) model augmented with multi-head temporal attention. In a similar vein, Dong et al. [126] recommended the application of pre-trained models for the extraction of deep Speaker Recognition (SR) and Speech Emotion Recognition (SER) features. Their approach synergizes these two profound speech features to capture the complementary data embedded within speaker voice characteristics and emotional variances.

**EEG.** The field of depression diagnosis has witnessed the burgeoning integration of electroencephalogram (EEG) and machine learning techniques, marking a pivotal research trajectory. In the reported literature [127], a novel deep learning method named the Asymmetry Matrix Image (AMI) is introduced, which constructs spatial distribution maps from EEG signals by assessing the asymmetry between cerebral hemispheres. AMI has been shown to outperform traditional methods, delivering superior classification accuracy and enhancing the distinction between depression patients and healthy controls. Additional research [128] delves into the utilization of nonlinear EEG signal features, such as Higuchi’s fractal dimension (HFD) and sample entropy (SampEn), which serve as indicators of signal complexity and irregularity. These nonlinear metrics have proven efficacious in segregating depression patients from healthy individuals, with high accuracy figures reported across a range of machine learning classifiers. In a different approach, literature [129] focuses on power spectral features and asymmetry measures within the alpha, beta, delta, and theta frequency bands. Notably, findings suggest that asymmetries in the alpha2 and theta bands, particularly when analyzed with a Support Vector Machine (SVM), lead to higher diagnostic precision, with an accuracy rate of 88.33%. Explorations into the use of EEG data for depression diagnosis have also extended to single-channel and multi-channel formats [130]. By refining feature selection and classification models via genetic algorithms, it has been discovered that single-channel analysis can effectively differentiate depression patients, underscoring the potential for employing portable EEG devices in preliminary depression screening despite a noted limitation in clinical generalizability due to small sample sizes. The literature [131] investigates four feature selection techniques and five classification algorithms for processing EEG data. Through rigorous data preprocessing and feature extraction—identifying noise types and harnessing both linear and nonlinear features—the critical role of the data preparation phase is emphasized for achieving optimal classification accuracy.

A novel article [47] presents a multi-modal feature fusion method that integrates EEG with eye movement (EM) signals, aiming to refine the identification of mild depression. The application of deep learning to fuse these multi-modal data sets enables real-time monitoring and detection of mild depression, with the fusion approach in the hidden layers yielding improved recognition accuracy over single-feature methods, and showcasing the benefits of combining diverse physiological signals. The melding of EEG and machine learning has advanced the diagnostic and treatment prediction capabilities for depression. Although challenges such as limited sample sizes and variability in feature extraction persist, forthcoming research endeavors are expected to tackle these issues, thereby enhancing the precision and utility of predictive models. Importantly, these advancements lay the groundwork for tailored treatment modalities, contributing to the delivery of more accurate and efficacious interventions for those suffering from depression.

**Multi-modal.** The landscape of depression diagnosis is rapidly evolving with the advent of multi-modal approaches, harnessing the rich data from speech, text, and video to create more nuanced and comprehensive diagnostic tools. Ehghaghi et al. [132] embarked on an interpretable analysis to discern the distinct characteristics between dementia and depression. They pinpointed a spectrum of differentiators such as auditory anomalies, repetitive speech patterns, word retrieval struggles, coherence degradation, and variance in lexical density and richness—all of which are pivotal in distinguishing these disorders. Diep et al. [133] ventured further by proposing a model that synthesizes deep learning features from both audio and text modalities, enriched with manually curated attributes deriving from domain expertise. Mao et al. [134] introduced a novel approach using an attention-based multi-modal framework to generate a joint speech and text representation, specifically for the prediction of depression. Exploring the intersection of speech and video modalities, Jan et al. [135] investigated the capability of cognitive machines and robots to autonomously recognize psychological states. By analyzing gestures and facial expressions, these intelligent systems aim to play a role in monitoring depressive states. Uddin et al. [136] optimized the data processing workflow by segmenting audio and video into fixed-length units for input into a spatiotemporal network. This network is tailored to extract both spatial and temporal characteristics, with the introduction of dynamic feature descriptors like the Volume Local Directional Structure Pattern (VLDSP) to capture the nuances of facial dynamics.

Not content with dual-modal analyses, some studies have ambitiously integrated all three modalities—speech, text, and video—to push the boundaries of depression detection. Yang et al. [137] contributed to this growing body of work by discussing a multi-modal depression analysis framework comprising deep convolutional neural networks (DCNNs) and deep neural networks (DNNs). This composite approach leverages the strengths of each modality, offering a more robust and potentially accurate detection system. The convergence of such diverse modalities represents a significant step forward in the field of mental health diagnostics. By combining distinct but complementary data sources, these integrated approaches aim to mirror the complex nature of depression more closely, offering promising directions for future research and potential clinical applications. The ultimate goal is to refine these tools for enhancing early detection and personalizing treatment strategies, thus providing a beacon of hope for individuals grappling with depression.

### 3.4. Diagnosis of Heart Disease

Heart diseases, particularly Cardiovascular Diseases (CVD), stand as the leading cause of death worldwide. Hypertrophic Cardiomyopathy (HCM) poses significant challenges due to the thickening of the left ventricular walls of the heart. The modern era has seen a paradigm shift in heart disease diagnosis, leveraging advanced technologies across various modalities. This chapter will diagnostic methods for heart disease using hypertrophic cardiomyopathy (HCM) as an example. We will gain a deeper understanding of HCM-assisted diagnostic techniques based on echocardiography, medical text data, and electrocardiograms (ECG) and explore other heart disease diagnostic methods based on genetic data. The comprehensive application of these diagnostic tools provides support for the early identification and treatment of heart disease and is of great significance for improving patient prognosis and quality of life. This section provides a summarized overview of the model and its features, as detailed in the accompanying Table 6.

**Echocardiography.** Deep learning frameworks have shown remarkable promise in enhancing the accuracy and efficiency of heart disease detection and classification. Among these advancements, the work of Almadani et al. [138] stands out with the introduction of the HCM Dynamic Echo, an end-to-end deep learning framework designed for the binary classification of echocardiography videos into hypertrophic cardiomyopathy (HCM) or normal categories. This system includes two analytical components: Branch 1, dubbed the Slow Path, which focuses on extracting spatial features, and Branch 2, known as the Fast Path, which is dedicated to capturing temporal structure information, thereby improving the accuracy of video recognition. They applied transfer learning and pre-trained HCM Dynamic Echo on the large Stanford EchoNet Dynamic Echocardiography dataset, enabling HCM detection in smaller echocardiography video datasets. In rigorous evaluations, HCM Dynamic Echo outperformed state-of-the-art baselines, with an accuracy of 93.13%, an F1 score of 92.98%, a Positive Predictive Value (PPV) of 94.64%, a specificity of 94.87%, and an Area Under the Curve (AUC) of 93.13%.

Parallel to these developments, other researchers have also made significant contributions to the field. For instance, Madani et al. [139] developed a high-efficiency deep learning classifier for binary Left Ventricular Hypertrophy (LVH) diagnosis using echocardiography images. The core framework of their model included a U-Net for eliminating auxiliary information from image and a series of convolutional neural networks, resulting in an accuracy of 91.2%. To counter data scarcity, they proposed data augmentation using semi-supervised Generative Adversarial Networks (GANs). GANs demonstrated superior performance than traditional CNNs with limited data, attaining a test accuracy of 92.3%. Nasimova et al. [140] introduced a deep convolutional neural network for classifying echocardiography videos as Dilated Cardiomyopathy or Hypertrophic Cardiomyopathy. Their study initially generated an Echo dataset from internet-sourced Echo videos and EchoNet database videos. The team trimmed the collected videos to 2–5 s to remove unnecessary echo information and redundant frames before segmenting them into 112 × 112 × 3 images for manual feature extraction. These images and extracted features were input into a six-layer CNN for classification, achieving a test accuracy of 98.2%.

Moreover, some studies have contributed to the field by applying deep learning models to diagnose various cardiac conditions from echocardiography. Zhang et al. [141] utilized the VGG-16 model to automatically detect three diseases from echocardiography: Hypertrophic Cardiomyopathy, Pulmonary Arterial Hypertension, and Cardiac Amyloidosis. They trained separate networks for each disease, using three random images per video. The images were processed through the VGG-16 model with a fully connected layer featuring two output units, achieving an AUC of 93% and *p*-value of 0.23 for HCM detection. Ghorbani et al. [142] analyzed 3312 consecutive comprehensive non-stress echocardiography studies collected from June to December 2018. The process started with the first frame of each video, sampling 20 frames at intervals of 100 milliseconds. The Inception-Resnet-v1 network processed each frame individually, and the final prediction was determined by averaging the predictions from all individual frames. This method achieved an AUC-ROC of 0.75 and an F1 score of 0.57.

**Medical text data.** Sundaram et al. [143] developed a Random Forest (RF) model to automatically identify patients with Hypertrophic Cardiomyopathy (HCM) using features extracted from Cardiac Magnetic Resonance (CMR) imaging reports. The Random Forest (RF) model attained an accuracy of 86% using 608 features and achieved 85% accuracy with 30 features. Mishra et al. [144] introduced an innovative application within the medical Internet of Things (IoMT) domain. They utilized a Recurrent convolutional neural network (Rec-CONVnet) to accurately estimate the risk of heart disease. The system design compiles various data points such as age, gender, symptoms of chest discomfort, blood sugar levels, blood pressure (BP), and other relevant clinical factors. Through comprehensive simulations and evaluations, the Rec-CONVnet demonstrated remarkable performance, achieving an impressive F1 score of 97%. Jayasudha et al. [145] designed a Social Water Cycle Driving Training Optimization (SWCDTO) ensemble classifier for heart disease detection. The classifier showed outstanding performance across specificity, accuracy, and sensitivity, reaching 95.84%, 94.80 and 95.36% in each metric. Levine et al. [146] investigated the performance of a large model (GPT-3) in diagnosing and triaging diseases like heart disease. The findings indicated that GPT-3’s performance nearly approached that of professional medical practitioners.

**Genetic data.** Peng et al. [147] employed a Support Vector Machine (SVM), Random Forest (RF), and Logistic Regression (LR) to develop a classification model for coronary atherosclerosis heart disease (CAD). This model utilized datasets GSE12288, GSE7638, and GSE66360 from the GEO database. The ROC curve analysis revealed for SVM, RF, and LR in validation to be 75.58%, 63.57%, and 63.95%, respectively. Their respective areas under the curve were 81.3% (95% CI 0.761–0.866, *p* < 0.0001), 72.7% (95% CI 0.665–0.788, *p* < 0.0001), and 78.3% (95% CI 0.725–0.841, *p* < 0.0001). Liu et al. [148] created a classification model for Coronary Artery Disease (CAD) using LASSO logistic regression, random forest, and SVM. They used data from the GEO dataset GSE113079, achieving an AUC of 97.1% in the training set and 98.9% in the testing set. Zhang et al. [44] introduced the Integration Machine Learning (IML) algorithm, incorporating a SVM, neural network (NN), RF, gradient boosting machine (GBM), decision trees (DT), and LASSO. This algorithm was applied to classify patients with Acute Myocardial Infarction (AMI) and stable coronary artery disease (SCAD), using GEO datasets GSE60993, GSE62646, GSE48060, and GSE59867, achieving an AUC over 90%. Hou et al. [149] utilized SVM for classifying CAD without heart failure (CAD-non HF), CAD complicated with heart failure (CAD-HF), and healthy controls, using GEO datasets GSE20681 and GSE59867. The study achieved an AUC of 0.944. Finally, Samadishadlou et al. [150] applied SVM for classifying myocardial infarction (MI), stable CAD, and healthy individuals, using datasets GSE59867, GSE56609, and GSE54475 from GEO. Their model demonstrated an AUC-ROC of 96% and an accuracy of 94%.

**Electrocardiogram.** The integration of Convolutional Neural Networks (CNN) into the analysis of Electrocardiogram (ECG) data has marked a significant leap forward in detecting Hypertrophic Cardiomyopathy (HCM) and other cardiovascular diseases (CVDs) [151]. Among the notable contributions, Tison et al. [152] developed an automated and highly interpretable method for analyzing patient ECG features. This method processed and analyzed 36,186 ECG datum from the University of California, San Francisco (UCSF) database. Researchers utilized Hidden Markov Models (HMM) to extract ECG vector representations containing 725 features, which were then trained using CNNs to estimate cardiac structural and functional indices and classify diseases. Compared to traditional neural network models, this vectorized processing approach better retained meaningful features in ECGs, thus enhancing the interpretability and accuracy of diagnostic results. Similarly, Dai et al. [151] used a deep CNN to classify five cardiovascular diseases (CVDs) using standard 12-lead ECG signals. The study utilized the public Physiobank (PTB) ECG database. The researchers have segmented ECG signals into different intervals—1 s, 2 s, and 3 s—without detecting individual waves, thus forming three distinct datasets. They applied ten-fold cross-validation on one-second-long ECG signals and tested on the other two datasets (two and three seconds long). The proposed CNN model achieved an accuracy, sensitivity, and specificity of 99.59%, 99.04%, and 99.87%, respectively, for one-second signals, demonstrating superior performance. For two-second signals using pre-trained models, the system achieved an overall accuracy, sensitivity, and specificity of 99.80%, 99.48%, and 99.93%. For three-second signal detection, the accuracies were 99.84%, sensitivity 99.52%, and specificity 99.95%. These results indicate that the proposed system achieved high performance while maintaining simplicity and flexibility, suggesting its potential for real-time application in medical settings.

Furthermore, Tison et al. [153] highlighted the application value of AI-enhanced ECG (AI-ECG) in assessing disease states and treatment responses for obstructive HCM. The study noted that AI-ECG could extract more physiologically and pathophysiologically relevant information related to obstructive HCM from ECGs, surpassing traditional manual interpretation methods. Moreover, the study mentioned the potential of AI-ECG for remote monitoring through smartphone electrodes to assess disease states and treatment responses. The authors also foresaw the future application of this technology in medication adjustment and enhancing treatment safety.

Another impressive study is conducted by the Mayo Clinic [154]: they used digital 12-lead ECGs from 2448 diagnosed HCM patients and 51,153 age and gender-matched non-HCM controls to train and validate a CNN. The algorithm performed impressively in adult HCM patient ECG detection, with an AUC of 0.96, sensitivity of 87%, and specificity of 90%. The algorithm’s performance in a test of 300 children and over 18,000 age and gender-matched controls was equally impressive: the HCM detection model achieved an AUC of 0.98, sensitivity of 92%, specificity of 95%, Positive Predictive Value (PPV) of 22%, and Negative Predictive Value (NPV) of 99%. The study found that the algorithm generally performed better in the adolescent group than in the pediatric group.

**Table 6:** Summary of different medical features for heart disease diagnosis.

Literature	Feature Name	Modality	Dataset	Results
Almadani et al. [138]	Echocardiography	echocar- diogram videos	Stanford EchoNet- Dynamic echocardiogram dataset	ACC: 93.13%, F1-score: 92.98%, Positive Predictive Value (PPV): 94.64%, specificity: 94.87%, AUC: 93.13%
Madani et al. [139]	echocardiography	Original echocardiograms	Private dataset	92.3% accuracy: binary left ventricular hypertrophy classification
Nasimova et al. [140]	Echocardiography	Clipped echocardiogram video frames	(1) EchoNet database; (2) Echo videos from the Internet	ACC: 98.2% (dilated cardiomyopathy vs. hyper-trophic cardiomy-opathy (HCM))
Zhang et al. [141]	Echocardiography	Original echocardiograms	Private dataset	AUC: 0.93
Ghorbani et al. [142]	Echocardiography	Cropped echocardiogram regions (inside of the scanning sector)	Private dataset	AUC: 0.75
Sundaram et al. [143]	Word Embedding, Part of Speech (POS)	Text	CMR	86% (ACC) for 608 features and 85% (ACC) for 30 features
Mishra et al. [144]	Word Embedding	Text	Real clinical records in hospital databases	97% F1 score, FPR of 64.6%, accuracy of 96.4%, and accuracy of 76.2%
Levine et al. [146]	Multivariate Features	Text	Recruited participants	Brier score = 0.18 for disease, Brier score = 0.22 for triage
Peng et al. [147]	Gene-based	RNA-seq	GEO	SVM: 81.3% (ACC); RF: 72.7% (ACC); LR: 78.3% (ACC)
Liu et al. [148]	Gene-based	RNA-seq	GEO	Training: 97.1% (AUC), test: 98.9% (AUC)
Zhang et al. [44]	Gene-based	RNA-seq	GEO	90% (AUC)
Hou et al. [149]	Gene-based	RNA-seq	GEO	94.4% (AUC)
Samadishadlou et al. [150]	Gene-based	MicroRNA	GEO	96% (AUC), 94% (ACC)
Dai et al. [151]	End-to-end Auto-learned Features	ECG	Physiobank (PTB) Public Dataset	Accuracy: 99.84%, Sensitivity: 99.52%, Specificity: 99.95%
Tison et al. [152]	725 Features Extracted using Hidden Markov Models	ECG	UCSF Database	AUR: Range 0.94 to 0.77
Tison et al. [153]	End-to-end Auto-learned Features	ECG	UCSF Database	-
Ko et al. [154]	End-to-end Auto-learned Features	ECG	Public Mayo Clinic Developed Database	AUC: 0.96, Sensitivity: 87%, Specificity: 90%

### 3.5. Diagnosis of Epilepsy

Epilepsy, a prevalent neurological disorder affecting approximately 60 million people worldwide [155], poses significant diagnostic challenges. A range of symptoms characterizes it, and an effective diagnosis requires a multidisciplinary approach. This article explores various diagnostic methods employed in epilepsy detection, utilizing advanced technology and medical imaging. This chapter will explore auxiliary diagnostic techniques for epilepsy based on images, medical text data, and electroencephalography (EEG). These methods play a crucial role in improving the accuracy and efficiency of epilepsy diagnosis, providing us with a new perspective to understand this complex disease and bringing better medical services to patients. This section provides a summarized overview of the model and its features, as detailed in the accompanying Table 7.

**Medical video.** Using video data for computer-assisted diagnosis has become essential for the timely detection of epilepsy. Karácsony et al. [156] employed clinical Motion Capture (MoCap) to quantitatively analyze seizure-related symptoms such as ictal head turning and upper limb automatisms, marking a pioneering discovery in differentiating epilepsy syndromes, providing clinical localization and lateralization information. Maia et al. [157] applied a threshold-based approach to first detect regions of interest (beds) in video data, aligning them vertically for consistency, then utilized Convolutional Neural Networks and Multilayer Perceptrons to classify epileptic seizures, achieving 65% AUC. Achilles et al. [158] recorded 52 seizures at 15 frames per second using infrared and depth imaging sensors, training distinct Deep Convolutional Neural Network architectures (CNNs) on video frames (one CNN for infrared frames, another for depth frames). Combining outputs from both networks, they achieved the prediction of ictal or interictal epilepsy phases, with their method demonstrating high sensitivity (87%) and specificity (81%) for generalized tonic-clonic seizures.

Building upon these advancements, Ahmedt-Aristizabal [159] unveiled an innovative network approach that integrates 3D facial reconstruction with deep learning. The design of this approach aims to detect and measure orofacial semiotics in a collection of 20 seizure videos, featuring recordings from patients with temporal and extra-temporal lobe epilepsy. The developed network demonstrated its capability to differentiate between two types of epileptic seizures, achieving an average classification accuracy of 89%. It marks a significant advancement in computer vision and deep learning within non-contact systems, particularly for identifying common semiotics in real-world clinical environments. Significantly, this method departs from earlier epilepsy monitoring techniques by moving beyond the reliance on single-angle image information. In contrast, Kunekar et al. [160] proposed improving accuracy by utilizing information from multiple modalities instead of relying solely on features from a single viewpoint. Ahmedt-Aristizabal et al. [161] proposed a new modular, hierarchical, multi-modal system aimed at detecting and quantifying semiotic signs recorded in 2D monitoring videos. This method combines computer vision with deep learning architectures to learn semiotic features from facial, body, and hand movements.

**MRI.** MRI-generated 2D or 3D images enable a better understanding of the brain’s internal structure, pinpointing brain issues associated with epileptic seizures. fMRI has become indispensable tools in the detection and understanding of epileptic seizures by providing detailed images of the brain’s internal structure. Garner et al. [162] applied a machine learning approach using a Random Forest classifier, trained with resting-state functional MRI (fMRI) data, to predict epilepsy outcomes. The model achieved a 69% accuracy rate in predicting epilepsy outcomes on the test set after 100 stratified cross-validation rounds, using 70% of resting-state fMRI scans for training and 30% for testing. Similarly, Sahebzamani et al. [163] employed the Gram-Schmidt orthogonalization method alongside a unified tissue segmentation approach for segmenting brain tissues in MRI images. They calculated first-order statistical and Gray Level Co-occurrence Matrix (GLCM) texture features and trained SVM classifiers using features from either the entire brain or the hippocampus to diagnose epilepsy. This comprehensive segmentation and whole-brain analysis methodology yielded a 94% accuracy rate.

In the quest for early and accurate diagnosis, researchers like Si et al. [164] have turned to diffusion MRI techniques to detect subtle brain changes in conditions such as Juvenile Myoclonic Epilepsy. They emphasized the importance of early diagnosis in Juvenile Myoclonic Epilepsy (JME), a disorder that predominantly affects adolescents and poses significant developmental challenges. They utilized two advanced diffusion MRI techniques—High Angular Resolution Diffusion Imaging (HARDI) and Neurite Orientation Dispersion and Density Imaging (NODDI)—to create connectivity matrices that capture subtle white matter changes. By adopting transfer learning, they trained sophisticated Convolutional Neural Network (CNN)-based models for JME detection. Pominova et al. [165] explored various deep 3D neural architecture building blocks for epilepsy detection, using both structural and functional MRI data. They experimented with 12 different architectural variants of 3D convolution and 3D recurrent neural networks. Santoso et al. [166] proposed a novel integrated Convolutional Neural Network approach for classifying brain abnormalities (epilepsy vs. non-epilepsy) using axial multi-sequence MR images. The model comprised base learners with distinct architectures and lower parameter counts. By aggregating the outputs and predictions of these base models (through methods like majority voting, weighted majority voting, and weighted averaging) and feeding them into a meta-learning process with a SVM, they significantly enhanced the final classification performance.

**Medical text data.** Hamid et al. [167] showcased the potential to differentiate epileptic patients from those with psychogenic non-epileptic seizures (PNES). They developed an NLP tool based on an annotator modular pipeline to analyze electronic medical records, identifying grammatical structures and named entities. This algorithm was proficient in detecting concepts indicative of PNES and those negating its presence. Taking a different approach, Pevy and colleagues [168] utilized written records of conversations between patients and doctors to distinguish between epileptic seizures and PNES. They employed an NLP toolkit to extract specific features of speech formulation efforts, such as hesitations, reformulations, and grammatical repairs, from these transcripts. The algorithm then trained machine learning classifiers with these features, enabling it to distinguish patients based on their verbal expression patterns. Connolly et al. [169] further affirmed the effectiveness of NLP in differentiating among various epilepsy types, including partial epilepsy, generalized epilepsy, and unclassified epilepsy. By analyzing text features extracted from electronic medical records, their algorithm successfully classified different subtypes of epilepsy with remarkable accuracy.

**EEG.** Researchers frequently use CNN (Convolutional Neural Network) architectures, which can extract features automatically, unlike traditional machine learning classifiers that require manual extraction of features for detecting and classifying epileptic seizures effectively. Clarke et al. [170] developed a deep Convolutional Neural Network (CNN) for detecting epileptic seizure discharges, trained using a dataset comprising over 6000 marked events from a group of 103 patients diagnosed with Idiopathic Generalized Epilepsy (IGE). This newly proposed automatic detection algorithm showcased exceptional performance in identifying epileptic seizures from clinical EEGs. The system achieved an impressive average sensitivity of 95% and kept the average false positive rate to just one per minute. These results indicate that AI-powered computer-assisted EEG analysis could significantly improve the speed and precision of EEG assessments, thereby potentially enhancing treatment outcomes for epilepsy patients. Fürbass et al. [171] employed the Fast R-CNN method for object detection, using deep regression for localization estimation of EDs (negative peaks) and the UDA training process to handle noise and artefacts in EEG. The authors used EEG data from 590,000 epochs of 289 patients for unsupervised training and tested it against 100 proprietary datasets. The experimental results indicated that the DeepSpike algorithm attained a sensitivity of 89%, a specificity of 70%, and an overall accuracy rate of 80%, showcasing its high effectiveness in identifying EEG discharges. Thara et al. [172] used a two-layer stacked bidirectional Long Short-Term Memory (LSTM) technique for detecting epileptic seizures. The researchers built a model with two LSTM layers, dropout and dense layers, and trained and optimized it using activation functions such as sigmoid and softmax, achieving good results with an accuracy of 99.89% on the training set and 99.08% on the test set. Yao et al. [173] experimented with ten different and independently improved RNN (IndRNN) architectures, achieving the best accuracy with a 31-layer Dense IndRNN with attention (DIndRNN).

**Multi-modality.** Torres-Velázquez et al. [174] evaluated the performance of multi-channel deep neural networks in Temporal Lobe Epilepsy (TLE) classification tasks under single and combined datasets. They trained, validated, and tested several multi-channel deep neural network models using brain structural indices from structural MRI, MRI-based region of interest correlation features, and personal demographic and cognitive data (PDC). Results indicated that PDC alone provided the most accurate TLE classification, followed by the combination of PDC with MRI-based brain structural indices. These findings affirm the potential of deep learning methods, like mDNN models, in TLE classification when combined with multiple datasets.

**Table 7:** Summary of different medical features for epilepsy diagnosis.

Literature	Feature Name	Modality	Dataset	Results
Karácsony et al. [156]	Medical video	2D + 3D video feature	Neuro- Kinect	-
Maia et al. [157]	Medical video	Original Infrared video feature	Private data	0.65 (AUC)
Achilles et al. [158]	Medical video	infrared and depth video frames	ADNI, AIBL	sensitivity (87%) specificity (81%)
Ahmedt-Aristizabal et al. [159]	Medical video	Regions of interest by 3D face reconstruction from the original video sequences	Private dataset	0.89 (ACC)
Ahmedt-Aristizabal [161]	Medical video	2D monitoring videos	Private dataset	83.4 % (ACC: face); 80.1% (ACC: body) body; 69.3% (ACC:hand)e
Garner et al. [162]	MRI	functional magnetic resonance imaging (fMRI) data	REDCap	0.69 (ACC)
Sahebzamani et al. [163]	MRI	first-order statistical and volumetric gray-level co-occurrence matrix (GLCM) texture features from structural MRI data	Private dataset	0.94 (ACC)
Si et al. [164]	MRI	the connectivity matrix which can describe tiny changes in white matter	Private dataset	75.2% (ACC) and the 0.839 (AUC)
Pominova et al. [165]	MRI	3D + 4D MRI data	Private dataset	0.73 (AUC)
Santos et al. [166]	MRI	axial multi-sequences of MRI	Private dataset	86.3% (ACC) 90.75% (F1-score)
Hamid et al. [167]	stemming features, POS, bag of concepts	Text	VA national clinical database	The accuracy, sensitivity, and F-score are 93%, 99%, and 96%
Pevy et al. [168]	Word embedding	Text	Recording, transcribing, and writing records of interview corpora	71% (ACC)
Connolly et al. [169]	N-gram	Text	DrWare- house (DrWH)	0.708 (F1) for partial epilepsy (PE), generalized epilepsy (GE), and unclassified epilepsy (UE), 0.899 (F1) for PE and GE
Clarke et al. [170]	End-to-end Auto-learned	EEG	Public Ad-hoc	Average Sensitivity: 95%
Fürbass et al. [171]	End-to-end Auto-learned	EEG	Private Dataset (Test); 590,000 Epochs from 289 Patients in Temple University’s Public EEG Corpus (Training)	Sensitivity: 89%, Specificity: 70%, Overall Accuracy: 80%
Thara et al. [172]	End-to-end Auto-learned	EEG	Private Dataset	Accuracy: 99.89%
Yao et al. [173]	End-to-end Auto-learned	EEG	CHB-MIT Dataset	Average Sensitivity: 88.80%, Specificity: 88.60%, Precision: 88.69%
Torres-Velázquez et al. [174]	Multi-modality	brain structure metrics from structural MRI, MRI-based region of interest correlation features, and personal demographic and cognitive data (PDC)	Private Dataset	Acc = 69.46% ± 20.82%, AUC = 70.00% ± 26.00%

### 3.6. Discussion

**Modality distinction.** In our comprehensive review, we examine the different methods used to automatically diagnose five specific diseases: Alzheimer’s disease (AD), breast cancer, depression, heart disease, and epilepsy. The medical data produced from different disease diagnosis processes has commonalities, mainly encompassing image, text, genetic, signal, and voice modalities. Distinctive preferences for specific modalities exist across different diseases. Even within the realm of single medical imaging, nuanced differences become apparent. For Alzheimer’s disease diagnosis, Magnetic Resonance Imaging (MRI) and Positron Emission Tomography (PET) images emerge as the predominant modalities, supplemented by the inclusion of voice data. The widespread use of MRI and PET stems from their effectiveness in capturing the structural and functional brain changes associated with Alzheimer’s disease (AD). The unique characteristics of neurodegenerative alterations make these imaging modalities particularly suitable for early detection and monitoring of disease progression.

Contrastingly, in breast cancer diagnostics, a multifaceted approach involves genetic data, X-ray imaging, ultrasound, and a notable amount of textual information. The rationale behind this approach lies in the heterogeneity of breast cancer itself, necessitating a comprehensive analysis of genetic predispositions, coupled with various imaging techniques and textual data to enhance diagnostic accuracy. Each modality contributes valuable insights into different aspects of breast cancer pathology, collectively enhancing the overall diagnostic efficacy. In the context of depression diagnosis, the emphasis shifts toward textual data and Electroencephalogram (EEG). The reliance on text data could be attributed to the subjective nature of depression symptoms, requiring a nuanced analysis of linguistic patterns and sentiment. EEG captures brain wave activity and complements textual data by providing physiological markers that indicate depression.

For heart disease diagnosis, the prevalent modalities include echocardiography, electrocardiography, and medical texts. The dominance of ultrasound-based echocardiography comes from its ability to provide real-time images of the heart’s structure and function, which is essential for assessing cardiac health. Electrocardiography contributes information on the heart’s electrical activity, while medical texts further contextualize the diagnostic process. For epilepsy diagnostics, a comprehensive strategy incorporates Magnetic Resonance Imaging (MRI), video data capturing patient movements, Electroencephalogram (EEG), and relevant textual information. The utilization of these diverse modalities is driven by the intricate nature of epilepsy itself, demanding a thorough examination of various aspects. MRI provides structural insights, video data offers observations of seizures and associated movements, EEG captures electrical activity in the brain, while textual information contributes contextual details.

In conclusion, the selection of modalities for automated diagnosis is intricately tied to the unique characteristics and pathological features of each disease. Understanding the rationale behind the prevalence of specific modalities facilitates a targeted and effective approach to automated disease diagnosis.

**Modality fusion.** Contemporary diagnostic methodologies increasingly favour the integration of multi-modal approaches. The advantages of the multi-modal paradigm lie in its ability to provide a more comprehensive and accurate understanding of complex phenomena by integrating diverse data modalities. This approach enhances robustness, improves interpretability, and allows for personalized and optimized solutions across various domains.

In diagnosing Alzheimer’s Disease (AD), where subtle but significant changes in language patterns and cognitive function are markers, combining speech and text analysis is extremely valuable. This multi-modal approach adeptly captures the intricate linguistic nuances and potential confusion in communication exhibited by AD patients. Integrating genetic data and electroencephalogram (EEG) as supplementary information enriches the diagnostic process, addressing the multifaceted nature of AD symptoms and facilitating a more accurate and holistic understanding. In cancer research, there is a significant emphasis on combining imaging and genetic data. Since genetic mutations play a pivotal role in the development and progression of various types of cancer, identifying specific genetic alterations associated with different types of cancer can provide insights into their molecular mechanisms and potential therapeutic targets.

Besides, specific genetic mutations may present as unique visual patterns. For example, specific genetic alterations in breast cancer, such as those in the BRCA genes, may result in characteristic radiographic features observable in mammograms or other imaging modalities. Therefore, combining genetic data with medical imaging enhances our molecular-level understanding of cancer and supports the creation of tailored, accurate methods for its diagnosis and treatment. Depression diagnosis predominantly relies on speech modalities, with supplementary integration of text or video data. This emphasis on speech is justified by the distinct changes in vocal patterns and tone often exhibited by individuals with depression. Adding text or video data enhances the diagnostic process by providing extra information on the patient’s emotional and behavioural conditions.

For diagnosing heart disease, it’s common to combine ultrasound imaging with medical texts. The rationale behind this lies in the need to comprehensively assess both structural and functional aspects of the heart. Ultrasound provides real-time visualizations of cardiac anatomy, while medical texts offer additional clinical context, creating a synergistic diagnostic approach. Epilepsy diagnosis currently benefits from the mutual utilization of various imaging modalities, such as Magnetic Resonance Imaging (MRI) and Positron Emission Tomography (PET) images. This approach acknowledges the diverse epileptic manifestations and leverages the strengths of multiple imaging techniques to achieve a more comprehensive and accurate diagnosis. In essence, the choice of modalities for fusion explicitly correlates with the diverse manifestations of patients’ conditions. The reasonable multi-modal fusion approach can capture the intricacies of symptoms, ensuring a more nuanced and effective diagnostic outcome tailored to the specificities of each medical condition.

**Performance improvement.** The evolution of research in automated disease diagnosis is accompanied by the continual improvement of performance. This progression has transitioned from machine learning dominance to primary reliance on deep learning, complemented by innovative techniques such as attention mechanisms and transfer learning. Initially, disease diagnosis methods focused on developing feature engineering within machine learning studies, where manually identifying and selecting pertinent features was vital for the model’s performance. However, this process had limitations, often requiring domain expertise and not fully exploiting the richness of complex datasets. In response to these challenges, the subsequent embrace of deep learning has become a transformative force in medical diagnostics. The distinctive advantage of deep learning lies in its capability to automatically extract hierarchical and intricate features from raw data, eliminating the need for explicit feature engineering. This automated feature extraction significantly enhances the diagnostic model’s performance by allowing it to discern intricate patterns and relationships within the data.

Deep learning has improved the accuracy and efficiency of disease detection. Within the domain of deep learning for medical diagnostics, scholars have proposed innovative techniques to elevate model performance. Inspired by how we humans see, attention mechanisms in deep learning models allow a focus on areas within the data for better analysis. It mimics the human ability to prioritize relevant information, improving the model’s ability to capture subtle or critical features. Attention mechanisms have shown effectiveness in different medical imaging tasks, leading to diagnoses that are more precise and aware of the context. Transfer learning has also become a technique to overcome the issue of scarce medical data samples. In transfer learning, a model pre-trained on a large dataset, often from a related domain, is fine-tuned on a smaller target dataset, which is typically scarce in medical applications. This approach leverages the knowledge gained from the source domain to enhance the model’s performance on the target task, even when training samples are limited. Transfer learning has proven effective in scenarios where acquiring a large, labeled medical dataset is impractical, thus facilitating the development of robust diagnostic models. The evolution from traditional machine learning, reliant on explicit feature engineering, to deep learning, with its automated feature extraction capabilities, has significantly improved disease diagnosis models. Combining attention mechanisms with transfer learning highlights scholars’ dedication to enhancing model performance, improving interpretability, and tackling the problem of limited data in medical contexts. These advancements collectively contribute to the ongoing refinement and enhancement of state-of-the-art diagnostic systems.

**Large model application.** The emergence of large models in AI has revolutionized many industries, particularly in healthcare. These models, often trained on vast datasets, can analyze complex patterns that lead to more accurate and efficient disease diagnosis. With the increasing use of electronic health records and the integration of various data sources, medical institutions now have access to more information. This dataset comprises patient histories, symptomatology, and genetic profiles, among other details, offering a rich reservoir. Large models can analyze this data to discern patterns and correlations. Currently, most large-scale models in healthcare focus on text, analyzing medical records, discharge summaries, and other types of written data. However, there is potential for models to analyze additional forms of medical data, including images, voice recordings, genetic data, and physiological signals.

As technologies improve and datasets grow, we can expect to see more diverse applications of large models in healthcare. For example, image analysis models can process medical images such as X-rays or CT scans to detect diseases or lesions more accurately. The speech analysis model can process the patient’s speech records and extract useful information from them, such as the severity of symptoms or the development trend of the condition. Genetic analysis models can predict a patient’s response to specific drugs or disease risks based on their genomic data. The physiological signal analysis model can track the patient’s vital signs, like heart rate and blood pressure, identify any irregularities swiftly, and take appropriate action. Notably, some challenges need to be solved. One major challenge is data privacy. Training and refining large models necessitates significant data volumes, yet it is essential to safeguard the privacy and security of medical information. Creating strong encryption and access management systems is crucial for patient data. It’s imperative to address ethical considerations when integrating AI into healthcare practices. It is essential to ensure that AI algorithms do not discriminate against any particular group and that their use complies with ethical standards. Overall, the rise of large models in healthcare can contribute to improving patient outcomes and reduce the burden on the healthcare system in the future.

## 4. Challenges and Future Works

Despite the commendable achievements in artificial intelligence (AI) technology within the realm of disease diagnosis and analysis, it is crucial to acknowledge that notable limitations still prevail in many other facets. Exploring solutions to overcome these limitations emerges as a pivotal concern for the future trajectory of this field. Consequently, herein, we delineate the extant constraints and proffer potential resolutions to these challenges.

### 4.1. Medical Multimodality Data Imbalance

Typically, data imbalance encompasses two dimensions: the imbalance within classes in a single modality, and the distributional imbalances across different modalities. This aspect describes the unequal representation of various classes within a single data category. For instance, in an MRI dataset, there might be a notable discrepancy in the number of scans illustrating Alzheimer’s disease compared to scans indicative of normal conditions. For the latter, there is a disproportionate representation of data from one modality compared to others: There could be a surplus of imaging data yet a scarcity of genetic or textual data about Alzheimer’s diagnosis. Some strategies are needed to solve the problem of imbalanced samples:

**Transfer learning:** Leveraging pre-existing labelled datasets from related medical domains and applying transfer learning techniques can partially address the data scarcity. One can refine pre-trained models by fine-tuning them on smaller, specialized datasets that cater to specific diagnostic challenges.

**Synthetic data generation:** Employing techniques for generating synthetic data, where new data points are artificially created based on existing labelled samples, can augment the available dataset. This approach helps address limitations arising from insufficient data volume.

**Ensemble methods:** You can enhance the accuracy of a model by combining predictions from multiple weakly supervised models or by incorporating different sources of weak supervision. Ensemble methods help compensate for the lack of detailed annotations by aggregating diverse model outputs.

### 4.2. Weak Model Generalization Ability

The core technologies and algorithms of AI models designed for different diseases are typically general. For instance, a Convolutional Neural Network (CNN) has been widely applied in the diagnosis of AD [80], breast cancer [96], depression [121], heart disease [140], and epilepsy [158]. However, deploying AI models developed for specific diseases to other disease predictions often demonstrates limited generalization ability. The primary reason lies in the fact that AI diagnostic models tailored for a specific disease tend to focus exclusively on the features unique to the particular disease, overlooking broader patterns. Some state-of-the-art techniques can address this issue:

**Considering multi-centre cross-institutional data collection:** Encouraging healthcare institutions to collaborate on data collection is to create more diverse and representative datasets. Such collaborative efforts involve pooling data from various sources, encompassing different geographical locations, demographic profiles, and medical practices. Models trained on datasets with this heightened diversity are more likely to generalize effectively across a spectrum of patient populations and healthcare scenarios.

**Adversarial training:** Adversarial training involves the introduction of adversarial examples during model training. By exposing the model to perturbed or deceptive samples, it learns to become more robust and exhibits improved generalization performance when faced with unseen or unexpected data. This technique can fortify the model against variations in the input space, enhancing its adaptability to a broader range of medical scenarios.

**Reinforcement learning:** Reinforcement learning is a paradigm where an agent interacts with an environment to learn optimal decision-making strategies. In medical diagnosis, one can use reinforcement learning to develop policies that help the model make more generalized decisions across diverse contexts. Through trial and error, the model hones its ability to navigate complex environments and adapt its behaviour to new and varied scenarios.

### 4.3. Lack of Model Interpretability

AI has demonstrated tremendous potential in health and medicine, yet research on the interpretability of AI decision outcomes is limited. This review found that only 28 of the included studies directly or indirectly tackled the crucial aspect of interpretability. These studies sought interpretability through methods like logistic regression, decision trees, naive Bayes, and support vector machines, known for their inherent clarity, or by applying techniques such as incorporating prior knowledge and using attention mechanisms to improve model interpretability. However, regrettably, the majority of studies did not adequately consider this crucial factor. Future research directions urgently need to delve into the interpretability of artificial intelligence models, utilizing interpretable models to enhance trust in AI and assist clinical practitioners in making informed decisions [175,176], thereby promoting the better integration of these models into clinical practice. Some solutions may be leveraged to enhance model interpretability:

**Combining inherently interpretable model architectures.** Several models such as decision trees or linear models, can be integrated with machine and deep learning frameworks thus enhancing transparency. These models provide explicit rules and feature importance, making their decision-making process more understandable.

**Visual heatmaps generation.** Generating heatmaps is a common technique for visualizing the importance or activation of specific regions in data. For instance, gradient-based methods like guided backpropagation or gradient-weighted class activation mapping (Grad-CAM) can identify influential regions, revealing which parts of the input most significantly contribute to the output.

### 4.4. Data Privacy and Security

Ensuring data privacy and security has always been a critical issue awaiting resolution in medical artificial intelligence. The development of robust AI models relies on extensive training and validation datasets. Because local data is often scarce, it’s usually necessary to centralize the data. However, centralized solutions come with inherent drawbacks, including concerns about data ownership, confidentiality, privacy, and security, as well as the potential for data monopolies biased towards data aggregators [177]. Means to mitigate these pitfalls include:

**Anonymization and de-identification.** This method is primarily achieved by removing or blurring information in the data that identifies individuals, thereby reducing the link between the data and specific persons. This method is widely employed in current research to safeguard patient privacy. However, studies indicate that even desensitized data may still be re-identifiable through sophisticated analysis methods [178].

**Federated learning.** Federated Learning [179] is a decentralized learning approach that pushes the model training process to local devices, forming a global model through local updates, thereby preventing sensitive data from leaving the original devices. This method of decentralized learning emerges as a progressive approach to tackle the challenges of data anonymization and de-identification, offering a proactive strategy for maintaining data privacy and security.

**Swarm learning.** Swarm learning [180] extends the principles of federated learning to scenarios involving multiple participants, facilitating the integration of data from various sources through collaborative learning. This approach ensures a more comprehensive and accurate learning outcome while safeguarding privacy.

### 4.5. Ethical and Moral Considerations

From an ethical and moral standpoint, it is vital to guarantee that developed models mitigate “bias” and “inequality” across individuals and demographic categories. It is particularly crucial to address disparities linked to gender, age, race, income, education, and geographic location to promote fairness. In most studies reviewed, the persistence of differences often stemmed from not having enough data to achieve mitigation. However, for the deployment of AI models in clinical practice, ensuring fairness and generalizability [181,182] is also essential to guarantee the ethical and effective implementation of these technologies in a clinical setting [183].

There are at least two common scenarios where ethical issues arise in medical data. The first scenario is when the data source itself cannot reflect the true epidemiological situation within a given population, such as population data bias resulting from overdiagnosis of schizophrenia in African Americans [184]. The second scenario is when the dataset used for algorithm training lacks members from specific demographic groups. For example, an algorithm primarily trained on data from elderly white males might yield poor predictions for young black females. If algorithms trained on datasets with these characteristics are adopted in healthcare, they may exacerbate health disparities [185]. Effective solutions include:

**Balanced data sampling.** When constructing the training dataset, employ methods such as undersampling, oversampling, adaptive sampling, etc., to ensure a relatively balanced number of samples from different groups. This helps prevent the model from overly focusing on a specific population, thereby reducing data bias.

**Removal of sensitive attributes.** Eliminate potentially sensitive attributes (e.g., gender, race, age, etc.) from the data to ensure that the training dataset for the model does not contain direct or indirect ethical information.

**Establishment of best practices by scientific societies and regulatory bodies.** Scientific societies and regulatory bodies should develop data assessment standards, allowing datasets to comprehensively and accurately represent the societal, environmental, and economic factors impacting health [186]. The aim is to identify and minimize bias in training datasets, thereby fostering the development of algorithms that mitigate bias and promote fairness. As a notable example of bias reduction, the U.S. Food and Drug Administration (FDA), within the context of its Digital Health Innovation Action Plan, initiated a pre-certification pilot program. They evaluate developing medical software based on five established excellence principles, including quality standards and other similar regulatory criteria [187]. These standards can be extended to encompass the risk of bias in training datasets, thereby addressing issues related to data “bias” and “inequality”.

### 4.6. Future Works

**Application of AI on mobile devices.** Integrating AI programs on mobile devices injects a more efficient and intelligent element into the management of patient diseases, early warnings, and promotion of healthy behaviours [188,189]. Equipping various sensors and AI programs on devices such as watches and smartphones enables real-time monitoring, recording, and analysis of patients’ vital signs (such as heart rate, blood pressure, oxygen levels, etc.), medication usage, dietary habits, and exercise data. This capability facilitates patients’ current physical conditions and future trends, enabling timely responses to potential health risks and offering personalized treatment recommendations.

**Brain-machine interfaces.** Brain-machine interfaces (BMIs) [190] are poised to play a crucial role in the diagnosis of neurological disorders in the future. BMIs, through direct interaction with signals from the brain, hold the potential to identify diseases related to the nervous system, such as Parkinson’s disease or stroke. BMIs are anticipated to advance brain diagnostics, particularly in the field of neuroimaging.

**Collaboration of diverse teams.** The application of AI in the health and medical field involves three types of parties, i.e., healthcare professionals, researchers, and AI experts. Facilitating collaboration among these three parties contributes to the advancement of AI in the health and medical domain. Healthcare professionals possess rich clinical experience and specialized medical knowledge, providing profound insights into the pathology, physiology, and other aspects of diseases. They can offer unique perspectives and high-quality annotated data for researchers and AI experts, thereby contributing to more interpretable and accurate AI models for disease diagnosis. Secondly, healthcare professionals recognize the significance and delicate nature of medical data, as well as the need to maintain its privacy and security. They can ensure the privacy protection and compliance of data, ensuring that researchers and AI experts, in the process of refining AI models, mitigate bias and promote fairness. Reciprocally, researchers and AI experts possess proficient technical development experience, enabling them to provide healthcare professionals with adaptive AI models for the ever-evolving medical environment. These models assist healthcare professionals in clinical diagnosis, achieve early disease warning and prediction, and alleviate their workload.

## 5. Conclusions

In this paper, we thoroughly investigate the applications of artificial intelligence in diagnosing five distinct disorders: Alzheimer’s disease, breast cancer, depression, heart disease, and epilepsy. We describe commonly used datasets to illustrate the data foundation, considering numerous multimodality data sources. Subsequently, we demonstrate the data pre-processing, feature engineering process, classification model establishment, and performance evaluation metrics. These methods automatically transform original data into valuable information highly relevant to disease lesions, representing key steps for AI-based diagnosis tasks.

We report and analyze detailed efforts on different modality-driven diagnoses, highlighting diverse strategies employed to address the complexities of each disorder. For Alzheimer’s disease, we scrutinize the integration of multi-modal data such as neuroimaging, genetic markers, and cognitive assessments, emphasizing the intricate interplay between various diagnostic modalities. In the field of breast cancer, we explore imaging data from mammograms and genetic information, offering a nuanced understanding of the disease at both structural and molecular levels. Regarding depression, we investigate textual and speech data, revealing the potential of linguistic and acoustic cues in enhancing diagnostic accuracy. For heart disease, we focus on physiological signals and imaging data, providing a holistic approach to cardiovascular health assessment. Additionally, in the case of epilepsy, we meticulously examine the integration of electroencephalogram (EEG) data, showcasing the significance of real-time monitoring and data-driven insights.

Finally, we acknowledge that while AI technology has made certain achievements in the medical field, significant limitations remain in disease diagnosis applications. We describe challenges such as medical multimodality data imbalance, weak model generalization ability, and lack of model interpretability, providing corresponding solutions to guide future work. Overall, this review aims to offer a valuable resource for clinicians, researchers, and stakeholders involved in the dynamic landscape of AI in healthcare by providing a comprehensive overview of advances in multi-modality-driven AI disease diagnosis.

## Figures and Tables

**Table 1:** Definition of the confusion matrix in binary classification.

	Actual Outcome		
		Positive	Negative
Predicted Outcome	Positive	TP	FP
	Negative	FN	TN

**Table 2:** The definition of performance evaluation metrics (note that the N, $p_{i}$ and $y_{i}$ in equation Brier score represent the number of samples, the predicted result for sample *i*, and the observed result (true label) of sample *i*, respectively).

Metric	Definition
Accuracy (ACC)	ACC = (TP + TN)/(TP + TN + FP + FN)
Precision (P)	P = TP/(TP + FP)
Recall (R)	R = TP/(TP + FN)
F1-score (F1)	F1 = 2 × P × R/(P + R)
Specificity (Sp)	Sp = TN/(TN + FP)
Brier score	Brier score = (1/N) × $\sum {\left[\left(p_{i}-y_{i}\right)\right]}^{2}$

**Table 5:** Summary of different medical features for depression disease diagnosis.

Literature	Feature Name	Modality	Dataset	Results
Aragon et al. [58]	Word embedding, hashtag	Text	eRisk 2018 and 2019	0.79 (F1) for Anorexia, 0.58 (F1) for Depression
Verma et al. [113], Ghosh et al. [114]	Word embedding	Text	Twitter data collected by Twitter API	78% (ACC)
Xu et al. [115], Qi et al. [116]	Multiple characteristics	Text	Dreaddit, DepSeverity, SDCNL, CSSRS-Suicide	0.816 (ACC) for Dreaddit, 0.775 (ACC) and 0.756 (ACC) for DepSeverity, 0.724 (ACC) for SDCNL, 0.868 (ACC) and 0.481 (ACC) for CSSRS-Suicide
Liu et al. [117]	MFCC, PLP, FBANK, TDNN × vector, Resnet × vector, I-vector	Speech	CN-Celeb, Depression speech database-20	accuracy: 74.72%
Liu et al. [118]	Short-term energy (power), intensity, loudness, zero crossing rate (ZCR), F0, jitter, flicker, formants and mel frequency cepstral coefficients (MFCC)), linear prediction coefficient (LPC), line spectrum pair (LSP)), perceptual linear prediction coefficient (PLP), etc.	Speech	private dataset	78.02% Accuracy
Jiang et al. [119]	Prosodic, spectral, and glottal features	Speech	private dataset	The accuracy was 75.00% in women and 81.82% in men, and the sensitivity/specificity ratio was 79.25%/70.59% in women and 78.13%/85.29% in men
Liu et al. [120]	MFCC, LPC, Jitter, Fundamental Frequency, etc.	Speech	private dataset	The recognition accuracy for males and females was 75.8% and 68.5% respectively
Yin et al. [121]	MFCC	Speech	DAIC-WOZ, MODM	F1: 92.7, Recall: 92.7, Precision: 92.8
Tasnim et al. [122]	Spectral features, depth representation features	Speech	DAIC-WOZ	F1: 69%
He et al. [123]	eGeMAPS, MRELBP, raw waveform, spectrogram	Speech	AVEC2013, AVEC2014	AVEC2013: RMSE 9.0000, MAE7.4210; AVEC2014: RMSE10.0012, MAE 8.201
Dubagunta et al. [124]	original speech signal, Low profile filtered signal (LPF), Linear Prediction Residual Signal (LPR), Homomorphically filtered speech source signal (HFVS), Zero frequency filtered signal (ZFF)	Speech	AVEC2013, AVEC2014	RMSE: 8.549, MAE: 6.650, F1: 0.824
Zhao et al. [125]	ComParE, some frame-level features	Speech	DAIC-WOZ, MODM	This model improves 2.3% and 10.3% compared to the LSTM model in public databases
Dong et al. [126]	Depth representation features	Speech	AVEC2013, AVEC2014	MSE: 8.549, MAE: 6.650, F1: 0.82
Kang et al. [127]	Matrix image of asymmetric feature transformation of EEG	EEG	Public dataset HUSM	Accuracy 98.85%
Čukić et al. [128]	HFD and SampEn of EEG signals	EEG	Private dataset (23 patents)	average accuracy 90.24% 97.56%
Mahato et al. [129]	Combined characteristics of alpha, alpha1, alpha2, beta, delta and theta power and theta asymmetry (delta, theta, alpha, beta, alpha1, alpha2) and theta asymmetry (average theta asymmetry and paired theta asymmetry)	EEG	Public dataset	average accuracy 88.33%
Wan et al. [130]	The feature extraction methods of time domain, frequency domain, wavelet, and nonlinear analysis are used to extract features from the subband components corresponding to the EEG samples.	EEG	Private (Beijing Anding Hospital, 12 normal people, 23 patients)	accuracy 86.67%
Cai et al. [131]	The linear characteristics are as follows: peak, variance, dip, kurtosis, and Hjorth parameters. Nonlinear characteristics include C0 complexity, correlation dimension, Shannon entropy, Kolmogorov entropy, and power spectral entropy.	EEG	Private dataset: 152 depressed patients and 113 healthy subjects	accuracy 71.32%
Zhu et al. [47]	1760 features (22 EEG features × 5 frequency bands × 16 electrodes)	EEG	Public dataset Ad-hoc	accuracy 83.42%
Ehghaghi et al. [132]	The acoustic features comprise spectral and sound-related characteristics, such as statistical functions of Mel-frequency cepstral coefficients (MFCC), fundamental frequency (F0), and zero-crossing rate (ZCR). Text features include syntactic complexity, semantic complexity, and discourse coherence, among others.	Speech, text	Dementia- Bank, Healthy Aging, ADReSS, DEPAC+, AD Clinical Trial	F1: 0.89 ± 0.03
Diep et al. [133]	Handcrafted features provided by domain experts include acoustic features, semantic features, and lexical-syntactic features.	Speech, text	DEPAC	F1: 63.0%
Mao et al. [134]	For speech, the features encompass prosodic features (NAQ, QOQ, H1–H2, PSP, MDQ, Peaklope, Rd), voice quality features (F0, VUV), and spectral features (MCEP, HMPDM, HMPDD). In the realm of text, GloVe word vectors are utilized.	Speech, text	DAIC-WOZg	accuracy 95.80%
Jan et al. [135]	Visual feature extraction includes Local Binary Pattern (LBP), Edge Orientation Histogram (EOH), Local Phase Quantization (LPQ), and deep feature extraction using pre-trained models like VGG-face and AlexNet. For audio feature extraction, Mel-frequency cepstral coefficients (MFCC) are employed. Additionally, the feature dynamic historical histogram involves MHH.	Speech, video	AVEC2013, AVEC2014	MAE: 6.14 RMSE: 7.43
Uddin et al. [136]	raw wav, image	Speech, video	AVEC2013, AVEC2014	AVEC2013: MAE 6.92, RMSE 8.54; AVEC2014: MAE 6.75, RMSE 8.45
Yang et al. [137]	For speech, statistical features are extracted. In the domain of text, paragraph vectors are utilized. For video, the feature extraction involves Displacement Range Histogram (DRH).	Speech, text, video	DAIC-WOZ	RMSE: 5.974, MAE: 5.163

## Data Availability

Data are available in a publicly accessible repository. Please refer to Table A1.

## Abbreviations

The following abbreviations are used in this manuscript:

AI	Artificial intelligence
AD	Alzheimer’s disease
HCM	Hypertrophic cardiomyopathy
ECG	Electrocardiogram
EEG	Electroencephalograms
CT	Computed tomography
MRI	Magnetic resonance imaging
PET	Positron emission tomography
SVM	Support vector machine
RNN	Recurrent neural network
CNN	Convolutional neural network
ADNI	Alzheimer’s disease neuroimaging initiative
UKB	United kingdom biobank
TCGA	The cancer genome atlas
BUSI	Breast ultrasound images
GEO	Gene expression omnibus
HCM	Hypertrophic cardiomyopathy
WHO	World health organization
SCD	Sunnybrook cardiac data
ACDC	Automated cardiac diagnosis challenge
DAIC-WOZ	Distress analysis interview corpus-wizard of OZ
MODMA	Multi-modal open dataset for mental-disorder analysis
WHO	World health organization
DICOM	Digital imaging and communications in medicine
PNG	Portable network graphics
ICA	Independent component analysis
DWT	Discrete wavelet transform
RFE	Recursive feature elimination
PCA	Principal component analysis
LDA	Linear discriminant analysis
CRF	Conditional random fields
LR	Logistic Regression
NB	Naive bayes
DT	Decision tree
LSTM	Long short-term memory
LM	Large Model
GPT	Generative re-trained transformer
PaLM	Pathways Language Model
SAM	Segment Anything Model
GLM	General Language Model
TP	True Positive
FP	False Positive
TN	True Negative
FN	False Negative
ACC	Accuracy
Sen	Sensitivity
Sp	Specificity
P	Precision
AUC-ROC	Area Under the ROC Curve
TPR	True Positive Rate
FPR	False Positive Rate
H-FCN	Hierarchical Fully Convolutional Network
MIL	Multiple Instance Learning
MCI	Mild Cognitive Impairment
CN	Cognitively normal
GRU	Gated Recurrent Unit
MLP	Multilayer Perceptrons
ASR	Automatic Speech Recognition
MM-SDPN	Multi-modal Stacked Denoising Predictive Network
RVFL	Random Vector Functional Link
SNP	Single nucleotide polymorphism
MAADf	Multi-modal AD diagnostic framework
CAD	Computer-Aided Diagnosis
NN	Neural Network
BF	Benign Fibroadenom
BPT	Benign Phyllodes Tumor
BTA	Benign Tubular Adenoma
MDC	Malignant Ductal Carcinoma
MLC	Malignant Lobular Carcinoma
MMC	Malignant Mucinous Carcinoma
MPC	Malignant Papillary Carcinoma
LASSO	Least Absolute Shrinkage and Selection Operation
RFE	Recursive Feature Elimination
mRNA	Maximum Relevance Minimum Redundancy
BUSI	Breast Ultrasound Images
EDLCDS-BCD	Integrated Deep Learning Clinical Decision Support System
USI	Ultrasound image
CKHA	Chaos Krill Herd Algorithm
CSO	Cat Swarm Optimization
LLM	Large Language Model
ELRDD	Ensemble Logistic Regression Model for Depression Detection
SR	Speaker Recognition
SER	Speech Emotion Recognition
AMI	Asymmetry Matrix Image
HFD	Higuchi’s fractal dimension
SampEn	sample entropy
EM	Eye movement
VLDSP	Volume Local Directional Structure Pattern
DCNN	Deep convolutional neural networks
DNN	Deep neural network
Bi-LSTM	bidirectional long short-term memory
ZCR	Zero crossing rate
MFCC	mel frequency cepstral coefficient
LPC	linear prediction coefficient
LSP	line spectrum pair
PLP	perceptual linear prediction coefficient
LPF	Low profile filtered signal
LPR	Linear Prediction Residual Signal
HFVS	Homomorphically filtered speech source signal
ZFF	Zero frequency filtered signal
PTB	Physiobank
CVD	Cardiovascular Diseases
HCM	Hypertrophic Cardiomyopathy
IoMT	Internet of Things
Rec-CONVnet	Recurrent CONVolutional neural network
SWCDTO	Social Water Cycle Driving Training Optimization
SCAD	Stable coronary artery disease patients
CAD-non HF	CAD without HF
CAD-HF	CAD complicated with HF
MI	Myocardial infarction
UCSF	University of California, San Francisco
HMM	Hidden Markov Model
PTB	public Physiobank
AI-ECG	AI-enhanced ECG
PPV	Positive Predictive Value
NPV	Negative Predictive Value
POS	Part Of Speech
MoCap	Motion Capture
fMRI	functional MRI
GLCM	Gray Level Co-occurrence Matrix
JME	Juvenile Myoclonic Epilepsy
HARDI	High Angular Resolution Diffusion Imaging
NODDI	Neurite Orientation Dispersion and Density Imaging
PNES	psychogenic non-epileptic seizures
IGE	Idiopathic Generalized Epileps
DIndRNN	Dense IndRNN with attentio
IndRNN	Independently improved RNN
TLE	Temporal Lobe Epileps
PDC	Personal demographic and cognitive data
PE	Partial epilepsy
GE	Generalized epilepsy
UE	Unclassified epilepsy
TCGA	The Cancer Genome Atlas
ADNI	Alzheimer’s Disease Neuroimaging initiative
OASIS-3	Open Access Series of Imaging Studies-3
AIBL	Australian Imaging, Biomarker and Lifestyle
SCD	Sunnybrook Cardiac Data
SAFHS	San Antonio Family Heart Study
BUSI	Breast Ultrasound Images
GEO	Gene Expression Omnibus
TLGS	Tehran Lipid and Glucose Study
SCD	Sunnybrook Cardiac Data
ACDC	Automated Cardiac Diagnosis Challenge
DAIC-WOZ	Distress Analysis Interview Corpus-Wizard of OZ
MODMA	Multi-modal Open Dataset for Mental-disorder Analysis

## Appendix A

**Table A1:** Multi-modal datasets of diagnosis task for different disease.

Dataset	Year	Disease	Modality	Link
Alzheimer’s Disease Neuroimaging initiative (ADNI)	2003	AD	Image-based	https://adni.loni.usc.edu/ (accessed on 29 November 2023)
Open Access Series of Imaging Studies-3 (OASIS-3)	2019	AD	Image-based	https://www.oasis-brains.org/ (accessed on 29 November 2023)
Australian Imaging, Biomarker and Lifestyle (AIBL)	2006	AD	Image-based	https://aibl.org.au/ (accessed on 29 November 2023)
Sunnybrook Cardiac Data (SCD)	2009	AD	Image-based	https://www.cardiacatlas.org/sunnybrook-cardiac-data/ (accessed on 29 November 2023)
Automated Cardiac Diagnosis Challenge (ACDC)	2018	HCM	Image-based	https://www.creatis.insa-lyon.fr/Challenge/acdc/ (accessed on 29 November 2023)
Cardiac CT Segmentation Challenge	2020	HCM	Image-based	https://www.ub.edu/mnms/ (accessed on 29 November 2023)
Congenital Heart Disease (CHD)	2013	Heart disease	Image-based	https://www.data.gov.uk/dataset/f13fbd0e-fc8a-4d42-82ef-d40f930e4b70/congenital-heart-disease-chd (accessed on 29 November 2023)
AMRG Cardiac Atlas	-	Heart disease	Image-based	https://www.cardiacatlas.org/amrg-cardiac-atlas/ (accessed on 29 November 2023)
Multi-Ethnic Study of Atherosclerosis	2002	Heart disease	Image-based	https://www.cardiacatlas.org/mesa/ (accessed on 29 November 2023)
Breast Ultrasound Images (BUSI)	2018	Breast cancer	Image-based	https://scholar.cu.edu.eg/?q=afahmy/pages/dataset (accessed on 29 November 2023)
Breast Cancer Coimbra Dataset	2013	Breast cancer	Text-based	https://archive.ics.uci.edu/ml/datasets/ (accessed on 29 November 2023)
Oncoshare Breast Cancer Database	2016	Breast cancer	Text-based	https://med.stanford.edu/oncoshare.html (accessed on 29 November 2023)
I2B2 NLP Research Database	2014	Breast cancer, Heart disease, Depression	Text-based	https://www.i2b2.org/NLP/DataSets/Main.php (accessed on 29 November 2023)
MIMIC-III Critical Care Database	2012	Heart disease, Depression	Text-based	https://github.com/MIT-LCP/mimic-code (accessed on 29 November 2023)
eDiseases Dataset	2018	Breast cancer, Heart disease, Depression, AD	Text-based	https://zenodo.org/record/1479354#.Y8P4kexBy3I (accessed on 29 November 2023)
National Alzheimer’s Coordinating Center (NACC)	1999	AD	Text-based	https://naccdata.org/ (accessed on 29 November 2023)
UK Biobank database	2010	Breast cancer, Heart disease, AD, Depression	Text-based	https://www.ukbiobank.ac.uk/ (accessed on 29 November 2023)
DementiaBank	2003	AD	Text-based	https://dementia.talkbank.org/ (accessed on 29 November 2023)
SAHS	2020	Breast cancer, Heart disease	Text-based	https://www.ncbi.nlm.nih.gov/projects/gap/cgi-bin/study.cgi?study_id=phs001215.v3.p2 (accessed on 29 November 2023)
TLGS	1999	Heart disease	Text-based	https://endocrine.ac.ir/page/Tehran-Lipid-and-Glucose-Study-TLGS (accessed on 29 November 2023)
Acute Myocardial Infarction Dataset of World Health Organization (WHO)	2023	Heart disease	Text-based	http://www.who.int/ (accessed on 29 November 2023)
UCI machine learning repository	2023	Heart disease	Text-based	https://archive.ics.uci.edu/dataset/45/heart+disease (accessed on 29 November 2023)
Depression text dataset	2023	Depression	Text-based	https://www.Depression-texts.com/ (accessed on 29 November 2023)
The Cancer Genome Atlas (TCGA)	2006	Breast cancer	Gene-based	https://www.cancer.gov/ccg/research/genome-sequencing/tcga (accessed on 29 November 2023)
Gene Expression Omnibus (GEO)	2000	Breast cancer	Gene-based	http://www.ncbi.nlm.nih.gov/geo (accessed on 29 November 2023)
Online Mendelian Inheritance in Man (OMIM)	1966	Breast cancer	Gene-based	https://omim.org/ (accessed on 29 November 2023)
GenBank	1982	Breast cancer	Gene-based	https://www.ncbi.nlm.nih.gov/genbank/ (accessed on 29 November 2023)
Human Gene Mutation Database (HGMD)	1996	Breast cancer	Gene-based	http://www.hgmd.org/ (accessed on 29 November 2023)
Genome Aggregation Database (genoAD)	2016	Breast cancer	Gene-based	https://gnomad.broadinstitute.org/ (accessed on 29 November 2023)
Chinese Millionome Database (CMDB)	2017	Breast cancer	Gene-based	https://db.cngb.org/cmdb (accessed on 29 November 2023)
University of California, Santa Cruz (UCSC)	2000	Breast cancer	Gene-based	http://www.genome.ucsc.edu/ (accessed on 29 November 2023)
Distress Analysis Interview Corpus-Wizard of OZ (DAIC-WOZ)	2014	Depression	Speech-based	https://dcapswoz.ict.usc.edu/ (accessed on 29 November 2023)
Multi-modal Open Dataset for Mental-disorder Analysis (MODMA)	2020	Depression	Speech-based, ECG-based	http://modma.lzu.edu.cn/data/index/ (accessed on 29 November 2023)
Depression and Anxiety Crowdsourced corpus (DEPAC)	2023	Depression	Speech-based	https://www.mturk.com (accessed on 29 November 2023)
Bipolar Disorder Corpus	2018	Depression	Speech-based, ECG-based	https://www.aconf.org/conf_153173.html (accessed on 29 November 2023)
AVEC2014	2014	Depression	Speech-based, Image-based	http://avec2014-db.sspnet.eu/ (accessed on 29 November 2023)
AVEC2013	2013	Depression	Speech-based, Image-based	http://avec2013-db.sspnet.eu/ (accessed on 29 November 2023)
ADReSS	2020	AD	Speech-based	https://luzs.gitlab.io/adress/ (accessed on 29 November 2023)
AVEC2019	2019	Depression	Speech-based	https://www.ihp-lab.org/resources/ (accessed on 29 November 2023)
ADReSS-M	2023	AD	Speech-based, Text-based	https://2023.ieeeicassp.org/ (accessed on 29 November 2023)
ADReSSo	2021	AD	Speech-based	https://luzs.gitlab.io/adresso-2021/ (accessed on 29 November 2023)
The Carolinas Conversation Collection (CCC)	2011	AD	Speech-based, Image-based	https://www.degruyter.com/how-access-works (accessed on 29 November 2023)
ERP Core	2016	AD	EEG-based	https://osf.io/thsqg/ (accessed on 29 November 2023)
EEG Epilepsy Datasets	2016	Epilepsy	EEG-based	https://www.researchgate.net/publication/308719109_EEG_Epilepsy_Datasets (accessed on 29 November 2023)
CHB-MIT Scalp EEG Database	2010	Epilepsy	EEG-based	https://physionet.org/content/chbmit/1.0.0/ (accessed on 29 November 2023)
Kaggle	2018	Epilepsy	EEG-based	https://www.kaggle.com/code/harunshimanto/machine-learning-algorithms-for-epileptic-seizures (accessed on 29 November 2023)
EEG_128channels_ERP_lanzhou_2015	2015	Depression	EEG-based	http://modma.lzu.edu.cn/data/application/ (accessed on 29 November 2023)
ECG-ID Database	2014	Heart disease	EEG-based	https://physionet.org/content/ecgiddb/1.0.0/ (accessed on 29 November 2023)
Common Standards for Electrocardiography (CSE) database	1980	Heart disease	EEG-based	http://www.escardio.org/Pages/index.aspx (accessed on 29 November 2023)
European ST-T Database	2009	Heart disease	EEG-based	https://physionet.org/content/edb/1.0.0/ (accessed on 29 November 2023)
Sudden Cardiac Death Holter Database	2004	Heart disease	EEG-based	http://physionet.org/physiobank/database/sddb/ (accessed on 29 November 2023)
Bonn EEG time series database	2001	Epilepsy	EEG-based	https://www.ukbonn.de/epileptologie/ag-lehnertz-downloads/ (accessed on 29 November 2023)
Temple University EEG corpus	2000	Epilepsy	EEG-based	https://isip.piconepress.com/projects/tuh_eeg/html/downloads.shtml (accessed on 29 November 2023)
